# Osterix mRNA Enrichment in Small Extracellular Vesicles Derived From Osteogenically Induced ADSCs: A Promoter of Osteogenic Differentiation in BMSCs

**DOI:** 10.1111/jcmm.70353

**Published:** 2025-01-13

**Authors:** Zhaoquan Liang, Yuelin Wu, Junhao Bao, Qiang Xiao, Sidong Luo, Xinfang Liu, Yeyang Wang, Chao Xie, Li Zhang

**Affiliations:** ^1^ Department of Spine, Orthopaedic Center, Guangdong Second Provincial General Hospital Southern Medical University Guangzhou China; ^2^ Department of Joint and Orthopedics, Zhujiang Hospital Southern Medical University Guangzhou China; ^3^ Department of Spine, Orthopaedic Center, Guangdong Second Provincial General Hospital Jinan University Guangzhou China

**Keywords:** adipose mesenchymal stem cells, bone marrow stem cells, osteogenic differentiation, osterix, small extracellular vesicles

## Abstract

Osteogenic differentiation of bone marrow stem cells (BMSCs) is essential for bone tissue regeneration and repair. However, this process is often hindered by an unstable differentiation influenced by local microenvironmental factors. While small extracellular vesicles (sEVs) derived from osteogenically induced adipose mesenchymal stem cells (ADSCs) reportedly can promote osteogenic differentiation of BMSCs, the underlying molecular mechanisms remain incompletely understood. In this study, we investigated the mRNA expression profile of ADSC‐sEVs^+^ and explored the role of specific mRNAs in the osteogenic differentiation of BMSCs. We first validated the osteogenic induction activity of ADSC‐sEVs^+^ through both in vitro and in vivo experiments. Using reverse transcription polymerase chain reaction, we compared mRNA expression between ADSC‐sEVs^+^ and ADSC‐sEVs and further assessed the impact of specific mRNAs on the differentiation of BMSCs through a series of in vitro experiments. One of our key findings was that osterix mRNA was highly enriched in ADSC‐sEVs^+^, which significantly enhanced alkaline phosphatase staining and upregulated downstream osteoblastic markers in BMSCs. Both overexpression and knockdown experiments confirmed that osterix mRNA is a critical signalling molecule that facilitates the differentiation of BMSCs into osteoblasts through ADSC‐sEVs^+^. This finding expands our understanding of the molecular mechanisms underlying the osteogenic differentiation of BMSCs and offers a promising strategy for targeted osteoblastic differentiation in clinical applications.

## 1 Introduction

Bone marrow mesenchymal stem cells (BMSCs) are extensively used in tissue engineering and regenerative medicine because of their exceptional differentiation potential, immune regulatory properties, angiogenic capabilities, and nerve reparative abilities. While BMSC differentiation into osteoblasts plays a pivotal role in bone tissue regeneration and repair [1], its clinical application has been hindered by the instability of their differentiation. This differentiation is predominantly influenced by various local microenvironmental factors present in damaged tissues, such as growth factors, cytokines, and extracellular vesicle [2], and manipulating these factors may help achieve the targeted differentiation of tissue‐engineered BMSCs.

Small extracellular vesicles (sEVs) secreted by adipose mesenchymal stem cells (ADSC‐sEVs) have gained popularity as cell‐free regenerative therapeutic tools because of their accessibility, abundant availability, low immunogenicity, high stability, and minimal donor site morbidity [3]. ADSC‐sEVs can transfer proteins, nucleic acids, and other bioactive molecules through paracrine signalling, effectively participating in various biological processes, such as angiogenesis, neurogenesis, and osteogenesis for tissue regeneration [4, 5, 6, 7]. Osteogenically induced ADSC‐sEVs (ADSC‐sEVs^+^) promote the differentiation of BMSCs into osteoblasts, whereas sEVs secreted by non‐induced ADSCS do not exhibit significant osteoinductive effects [8]; thus, certain alterations in ADSC‐sEVs^+^ may contribute to their osteoinductive capacity. Nevertheless, the specific component of ADSC‐sEVs^+^ responsible for this effect and the mechanism underlying the osteogenic differentiation of BMSC remain elusive. Investigating this topic can facilitate the regulation of targeted BMSC differentiation in clinical applications.

The complex and diverse internal components of ADSC‐sEVs^+^—including proteins, nucleic acids, and other bioactive molecules—may be involved in the induction of osteogenic differentiation of BMSCs. Although miRNA‐mediated regulation of downstream target mRNA expression has been extensively studied [9], whether exogenous pathways can upregulate or downregulate targeted mRNAs in recipient cells remains unclear. Osteoblast lineage cells express key signalling targets involved in osteogenic differentiation, such as osteopontin (OPN), osteocalcin (OCN), runt‐related transcription factor 2 (RUNX2), osterix (also known as OSX or SP7), and nuclear factor of activated T‐cells c1 (NFATc1) [10]. The corresponding mRNAs encoding these factors are important signalling targets in the osteogenic differentiation process, with osterix playing a crucial role in regulating various osteogenic factors. Although its mechanism involves the modulation of intracellular signalling pathways, investigations on its potential as an extracellular signalling component in the osteogenic differentiation of other cell types are lacking. Considering that ADSC‐sEVs^+^ promoted the osteogenic differentiation of BMSCs, we speculated that these mRNAs might be related to ADSC‐sEVs^+^.

In the present study, we initially confirmed the positive osteoinductive effect of ADSC‐sEVs^+^ on BMSCs and subsequently investigated the presence of osterix mRNA in ADSC‐sEVs^+^. A key finding of our investigation was the high expression levels of osterix mRNA exhibited by ADSC‐sEVs^+^. Moreover, we postulated that osterix mRNA contributes to the osteoinductive properties of ADSC‐sEV ^+^. We also conducted gain‐and‐loss experiments using siRNA transfection for downregulation and rhBMP2 coculture for upregulation.

## 2 Materials and Methods

### 2.1 ADSCs and BMSCs Harvesting

Male New Zealand white rabbits aged 2–3 months (purchased from Guangdong Medical Laboratory Animal Centre) were sacrificed to obtain subcutaneous groin adipose tissue and extract femur‐knee‐tibia for mesenchymal stem cells (MSCs). The adipose tissue was cut into small pieces, digested with collagenase I (Sigma‐Aldrich, USA) solution, and filtered; subsequently, we isolated ADSCs by centrifugation at 1200 rpm for 10 min and precipitated the cells. During BMSC extraction, the epiphyses of the femur and tibia were rinsed with Dulbecco's Modified Eagle's medium (DMEM)/F‐12 (1:1) (Thermo Fisher Scientific, USA). The rinsed solutions were collected and centrifuged. We also collected the precipitated cells at the bottom of the centrifuge tube. The cells were then resuspended in DMEM/F12 containing 20% foetal bovine serum (FBS; Gibco, USA) and seeded in culture flasks. The culture medium was replaced according to cell growth, and culturing was continued until 80% fusion was achieved. All ADSCs and BMSCs used in subsequent experiments were at 2–4 passages. See Data S1 for further details.

### 2.2 Flow Cytometry Analysis of ADSCs and BMSCs

The cells isolated from the adipose tissue and bone marrow were characterised using rabbit‐specific markers for MSCs to confirm their phenotype. ADSCs and BMSCs at passage 2 were collected and incubated with MSC‐positive markers (CD44, CD90, CD29, and CD105) and MSC‐negative markers (CD34 and CD45). Subsequently, fluorophore‐conjugated secondary antibodies were used for incubation, and a non‐specific mouse or rat IgG secondary antibody was used as a negative control. Detailed information is presented in Data S1.

### 2.3 Osteogenic Differentiation and Identification

To test the osteogenic differentiation potentials, ADSCs were cultured in osteoblast differentiation medium (DMEM/F12 containing 10% FBS, 0.1 μM dexamethasone (Sigma‐Aldrich, USA), 10 mM β‐glycerol phosphate (Sigma‐Aldrich, USA), 50 μM ascorbic acid (Meilunbio, China), 100 IU/mL penicillin, and streptomycin), and the medium was replaced every 3 days. Osteoblast differentiation was assessed using alkaline phosphatase (ALP) and alizarin red S (ARS) assays. ALP activity was analysed to quantify ALP expression. sEVs derived from ADSCs after osteogenic induction for 14 days were denoted as ADSC‐sEVs^+^. Further details are provided in Data S1.

### 2.4 Isolation and Characteristics of ADSC‐sEVs

When the ADSCs reached approximately 90% confluence, the culture supernatant was replaced with DMEM/F12 medium containing 10% serum‐free FBS (Biological Industries, Israel) for 48 h. The conditioned medium was harvested from the ADSCs. Dead cells and cell debris were discarded by sequential centrifugation at 2000 × *g* for 20 min and 3200 × *g* for 10 min. The large extracellular vesicle (lEV) pellets were removed by ultracentrifugation at 20,000 × *g* for 30 min [11], and the sEV pellets were further collected by ultracentrifugation at 100,000 × *g* for 70 min at 4°C. Final sEVs were resuspended in phosphate‐buffered saline for immediate use or stored at −80°C. Purified sEVs were identified by transmission electron microscopy (TEM; Hitachi, HT‐7700, Japan). Size distribution was analysed using nanoparticle tracking analysis (NTA; Particle Metrix, Zeta VIEW, Germany). The concentration of sEVs was determined using an Enhanced BCA Protein Assay Kit (Beyotime, China). In addition, the sEVs markers, CD63 and TSG101, were detected using western blotting. The samples were stored at −80°C for RNA extraction or −20°C for protein analysis. Further details are provided in Data S1.

### 2.5 In Vitro Experiment Design

To explore the presence of target mRNAs (OPN, OCN, RUNX2, Osterix and NFATc1), their expression levels in ADSC‐sEVs were compared with those in ADSCs using reverse transcription polymerase chain reaction (RT‐PCR) analysis. Additionally, melting curve analysis was performed for each mRNA.

To test whether osteogenic induction caused osterix mRNA alteration in sEVs, osterix mRNA expression between ADSC‐sEVs and ADSC‐sEVs^+^ was compared.

The osteoinductive effect of ADSC‐sEVs^+^ on BMSCs differentiation was validated by adding them to BMSCs cultures followed by ALP staining and western blot analyses; ADSC‐sEVs were used as a control group. GW4869 (Abcam, USA) was used as an inhibitor to suppress the release of extracellular vesicles (EVs) from the ADSCs. Subsequently, samples obtained from the cell supernatant following an identical separation procedure for small EVs were labelled ADSC‐sEVs^+^(GW4869). The osteogenic differentiation potential of BMSCs cocultured with ADSC‐sEVs, ADSC‐sEVs^+^, and ADSC‐sEVs^+^(GW4869) was compared.

Immunofluorescence staining of BMSCs under basal conditions and coculture with ADSC‐sEVs^+^ at 15, 45, and 90 min confirmed their transport into BMSC by ADSC‐sEVs^+^. Additionally, RT‐PCR was used to compare osterix mRNA expression in BMSCs under basal conditions and coculture with ADSC‐sEVs^+^.

siRNA transfection was employed to knock down osterix mRNA expression in ADSCs, resulting in lower expression levels within ADSC‐sEVs^+^, which were verified using RT‐PCR and referred to as ADSC‐sEVs^+^(siRNA). Western blotting detected the impact on BMSCs osteoblast differentiation when cocultured with either unmodified or modified sEVs variants [ADSC‐sEVs^+^, ADSC‐sEVs^+^(siRNA)].

Conversely, rhBMP2 treatment upregulated osterix mRNA expression in ADSCs, leading to higher levels within ADSC‐sEVs^+^, identified as ADSC‐sEVs^+^(rhBMP2). Subsequent comparison revealed differences in their ability to induce osteoblast differentiation when cocultured with BMSCs.

### 2.6 RNA Extraction and Real‐Time Quantitative PCR

Total RNA was extracted from the cells using NcmZol Reagent (NCM Biotech, China) according to the manufacturer's guidelines. RNA was extracted from sEV fractions using an RNA Isolation Kit (RENGENBIO, China). All cell RNAs used for subsequent experiments had A260/A280 purity in the 1.9–2.1 range, whereas all the sEV RNAs were within the 1.0–1.5 range. Next, the mRNAs were reverse transcribed into cDNA using the NovoScript Plus All‐in‐one 1st Strand cDNA Synthesis SuperMix kit (Novoprotein, Suzhou, China). Subsequently, RT‐PCR was performed using a Roche LightCycler 480‐II PCR System (Roche, Switzerland) with a NovoStartSYBR qPCR SuperMix Plus kit (Novoprotein, Suzhou, China). The primers for OPN, OCN, Runx2, COL1, NFATc1, and osterix are listed in Table S3. Melting curves were generated to ensure the specificity of each qRT‐PCR. The relative expression levels of Osterix mRNA in each sample were calculated and quantified using the $\;2^{-△△\mathrm{Ct}}$ method after normalising to the expression of U6.

### 2.7 Western Blotting

Western blotting was performed on the protein lysates. Briefly, the proteins of EVs or cells were extracted with RIPA lysis buffer containing phenylmethylsulfonyl fluoride solution (Beyotime, China), quantified using the Enhanced BCA Assay Kit (Beyotime, China), separated by 10% sodium dodecyl sulphate‐polyacrylamide gel electrophoresis, and then transferred onto PVDF membranes. The membranes were immunoblotted with primary antibodies (Table S1). Protein bands were detected and analysed using an Alliance Q9 Micro Light Chemiluminescence Imaging System (UVItec Alliance, UK). Images were processed using the open‐access software ImageJ (NIH, USA). β‐actin and GAPDH were used as an internal control. Further details are provided in Figures S2–S5.

### 2.8 siRNA Transfection of ADSCs

siRNA was used to knock down osterix mRNA in the ADSC‐sEVs. ADSCs were cultured to 80%–90% confluence and transfected with several different groups of siRNAs (2.5 μg) (Table S2) for approximately 24 or 48 h. Transfection efficiency was analysed by observing the fluorescence expression. After determining the optimal siRNA‐lipid complex to maximise transfection efficiency, lysates and the corresponding media were used to analyse osterix mRNA expression in subsequent experiments. Further details are provided in Data S1.

### 2.9 Cellular Uptake Assay and Immunofluorescence

ADSC‐sEVs were labelled with the PKH67 Green Fluorescent Cell Linker Kit (Sigma‐Aldrich, USA) according to the manufacturer's instructions, and the labelled sEVs were used for the uptake experiments. We then cocultured these PKH67 sEVs with BMSCs. After the indicated time of coculture, we stained BMSCs with DAPI (Sigma Aldrich, USA) and observed them using confocal microscopy. Further details are provided in Data S1.

### 2.10 In Vivo Experiments

All animal experiments were performed in compliance with the guidelines of the EU Directive 2010/63/EU for animal experiments and approved by the Animal Ethics Committee of Guangdong Second Provincial General Hospital (Project Number: 2024‐DW‐KZ‐094‐02). Six female SPF‐grade rats, aged 5–6 months, were randomly assigned to three groups: the negative control (NC), ADSC‐sEVs, and ADSC‐sEVs^+^ groups. A tibial bone defect model was created for each rat under gas anaesthesia. Subsequently, the bone defect sites were injected with an equal volume of Matrigel, Matrigel containing 1.5 μg/μL ADSC‐sEVs, or Matrigel containing 1.5 μg/μL ADSC‐sEVs+ for the NC, ADSC‐sEVs, and ADSC‐sEVs^+^ groups, respectively. Three weeks postoperatively, micro‐CT scanning and histological analyses, including haematoxylin and eosin (H&E) and Goldner's trichrome staining, were performed to assess the bone regeneration in each group (Table S5). Further details are provided in Data S1.

### 2.11 Statistical Analysis

Statistical analyses were performed using GraphPad Prism 7.0 (GraphPad Software, USA). Data are shown as mean ± standard deviation of at least three independent experiments. Statistical differences were evaluated using a two‐sample unpaired *t*‐test if the two samples satisfied the homogeneity of variance or the Welch *t*‐test if not, with *p* values < 0.05 considered significant.

## 3 Results

### 3.1 Characterisation of ADSCs and BMSCs

**FIGURE 1:**
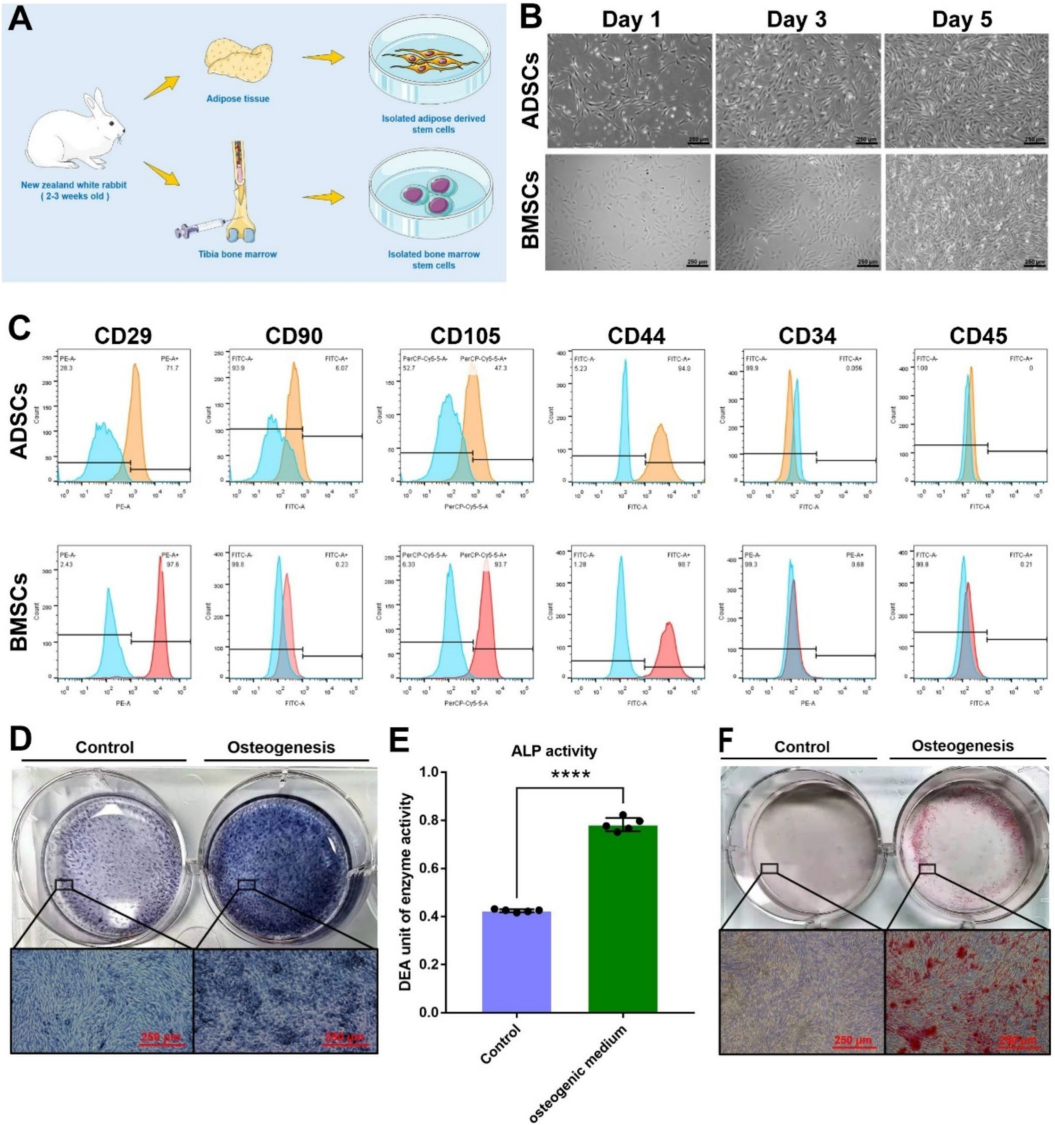
Characterisation of ADSCs and BMSCs. (A) Schematic illustration of ADSCs and BMSCs isolation and culture. (B) Morphological growth of ADSCs and BMSCs over a defined time course (scaler bar: 250 μm). (C) Flow cytometric analysis of different stem cell marker expressions in ADSCs and BMSCs. (D) ALP staining of ADSCs induced after 14 days of osteogenic induction (Scale bar: 250 μm). (E) ALP assay kit analysis of ADSCs induced by osteogenic induction medium for 14 days (Scale bar: 250 μm, *n* = 5, ****p < 0.05). (F) ARD staining of ADSCs induced by osteogenic induction medium for 21 days (Scale bar: 250 μm). ADSCs, adipose mesenchymal stem cells; ALP, alkaline phosphatase; ARD, Alizarin Red S; BMSCs, bone marrow stem cells.

The rabbits were sacrificed to obtain original tissues for ADSC and BMSC isolation (Figure 1A). ADSCs and BMSCs displayed a remarkable spindle‐shaped morphology (Figure 1B), with ADSCs generating more ALP deposits and calcified nodules under osteogenic stimulation, as evidenced by ALP and ARS staining (Figure 1D,F).

Flow cytometry analysis revealed that ADSCs and BMSCs were both positive for MSC surface‐specific markers, including CD29 (PE 71.7% and 97.6%, respectively), CD44 (FITC 94.8% and 98.7%, respectively), and CD105 (PerCP 47.3% and 93.7%, respectively), but were highly negative for haematopoietic stem cell surface markers (CD34 FITC, 0.056% and 0.68%, respectively; CD45 FITC, 0% and 0.21%, respectively; Figure 1C). However, CD90, an MSC‐specific surface antigen, showed only 6.07% and 0.23% positivity in ADSCs and BMSCs, respectively, consistent with the experimental data of some previous studies [12, 13, 14]. These features demonstrated that the isolated cells were in accordance with typical MSC characteristics. Characterisation of rat ADSCs and BMSCs is presented in Figure S1.

### 3.2 Characterisation of ADSC‐sEVs

**FIGURE 2:**
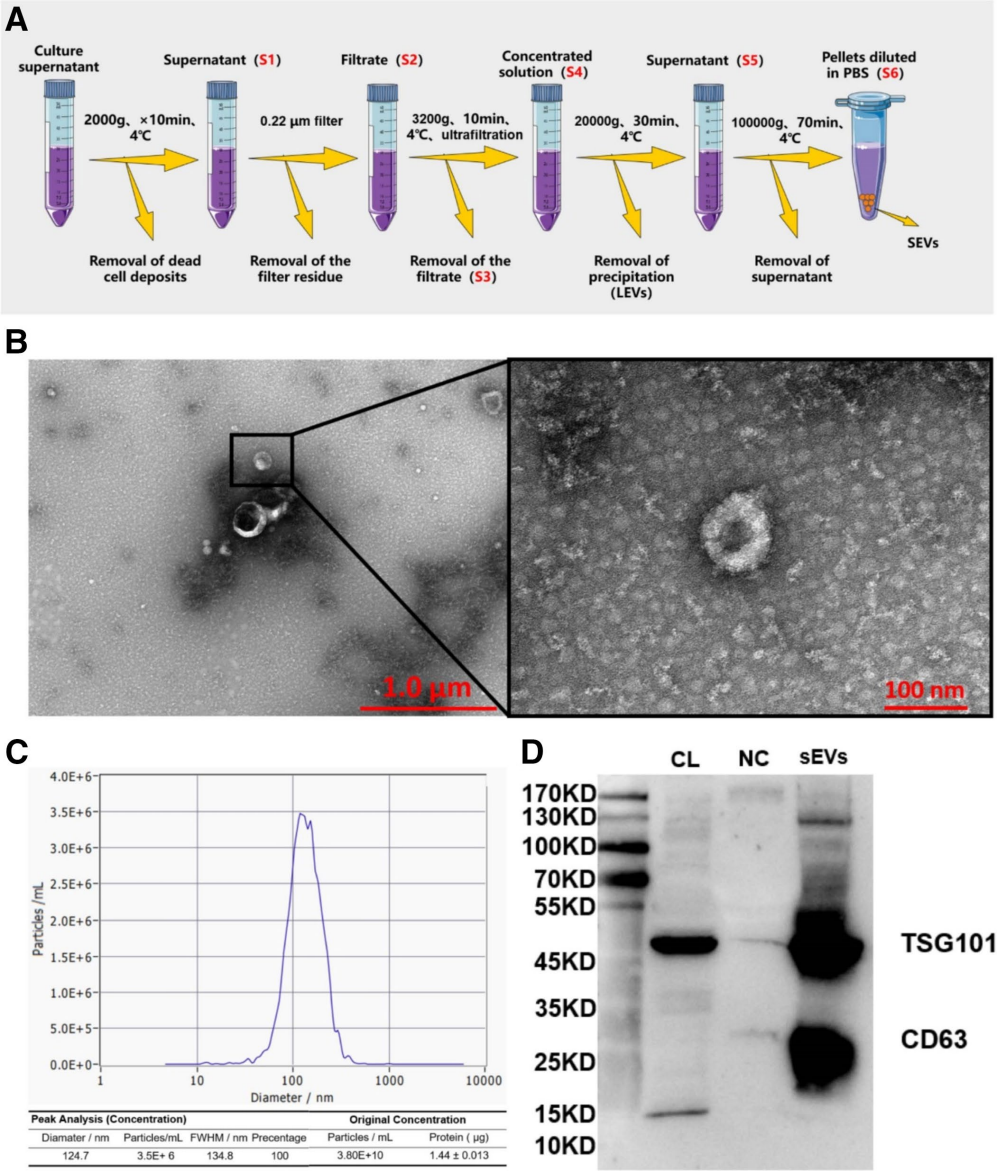
Characterisation of isolated ADSC‐sEVs. (A) Schematic overview of the sEVs isolation process from cell culture supernatant. (B) Representative transmission electron microscopy images depicting vesicular morphology with membrane enclosures (Scale bars: 1.0 and 100 nm). (C) Particle size distribution analysis via nanoparticle tracking, exhibiting a near‐normal distribution with a mean diameter of 143.1 nm. (D) Western blot detection of exosomal markers (CD63 and TSG101) in ADSC lysate (CL), PBS as a negative control (NC), and isolated sEVs. ADSCs, adipose mesenchymal stem cells; ALP, alkaline phosphatase; PBS, phosphate‐buffered saline; sEVs, small extracellular vesicles.

Adipose mesenchymal stem cells‐small extracellular vesicles were separated and purified using differential ultracentrifugation (Figure 2A). Transmission electron microscopic imaging exhibited particles' classical “cup‐shaped” structure, which were delimited by a lipid bilayer with a range of 50–150 nm in high‐magnification views (Figure 2B). Nanotracking analysis showed that these products had an original concentration of 3.8E+10 particles/mL, and the BCA method determined the protein concentration to be 1.446 μg/μL (Figure 2C). Both sEV‐specific phenotypic markers—CD63 and TSG101—were positive in ADSC‐sEVs compared to those in cell lysates, as evidenced by western blotting (Figure 2D and Figure S2). The survival rate of source cells secreting these sEVs was up to 99% (Table S4).

### 3.3 High Expression and Encapsulation of Osterix mRNA in ADSC‐sEVs^+^

**FIGURE 3:**
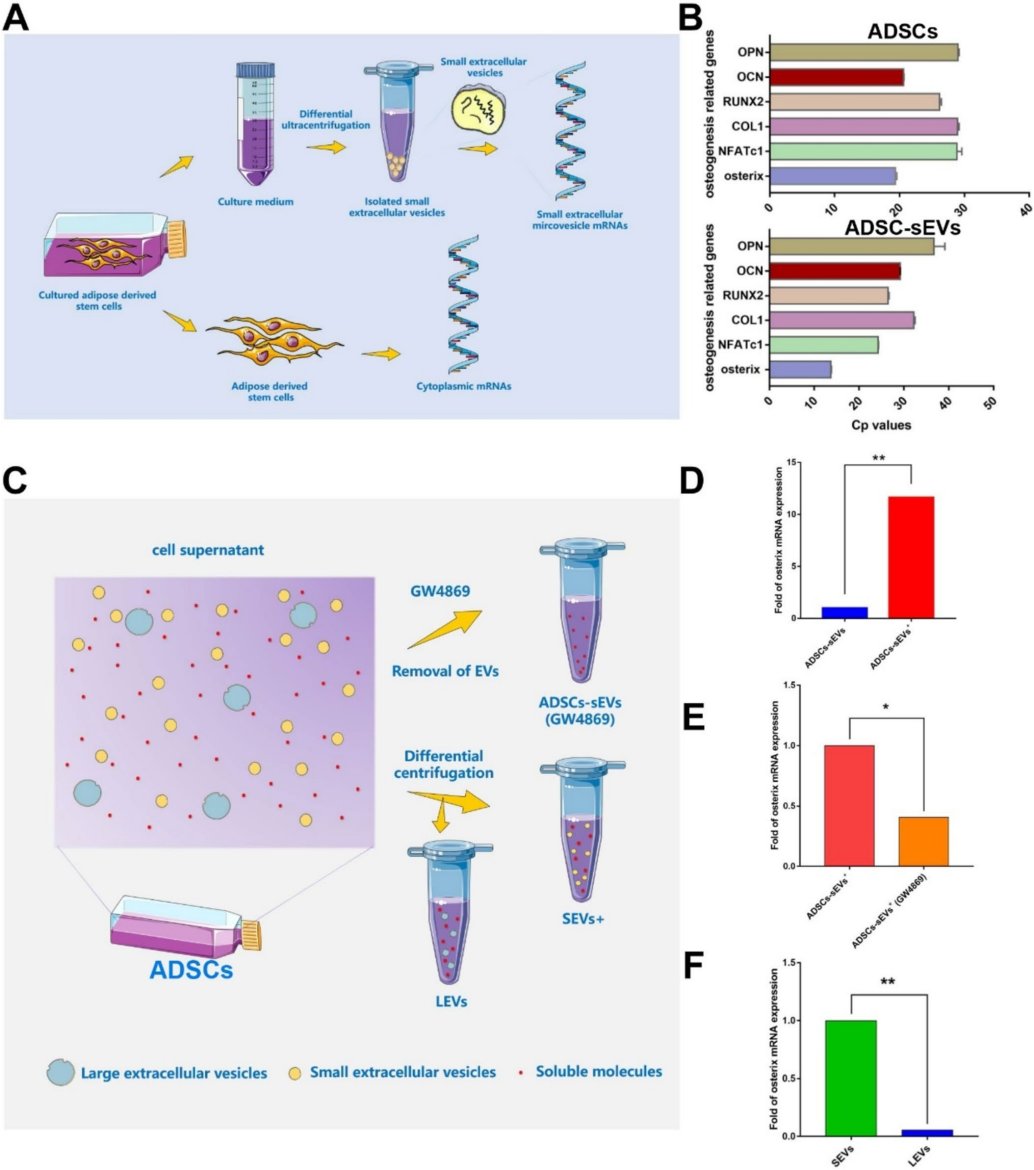
Osterix mRNA is highly expressed in ADSC‐sEVs^+^. (A) Schematic representation of mRNAs extraction from ADSCs and ADSC‐sEVs. (B) Levels of osteogenic mRNAs in ADSCs and ADSC‐sEVs (*n* = 4). (C) Components of ADSCs supernatant and methods used to distinguish them. (D) The expression of Osterix mRNA between ADSC‐sEVs and ADSC‐sEVs^+^ (*n* = 4, **p < 0.01). (E) The expression of Osterix mRNA between ADSC‐sEVs^+^ and ADSC‐sEVs^+^(GW4869) (*n* = 4, *p < 0.05). (F) The expression of Osterix mRNA between sEVs and lEVs (*n* = 4, **p < 0.01) ADSC‐sEVs^+^, sEVs derived from ADSCs with osteogenic induction; ADSC‐sEVs^+^(GW4869), sEVs derived from ADSCs with osteogenic induction and GW4869 treatment; ADSCs, adipose mesenchymal stem cells; lEVs, medium/large extracellular vesicles; sEVs, small extracellular vesicles.

mRNAs from ADSCs and ADSC‐sEVs were extracted to explore the differences in mRNA expression (Figure 3A). By analysing the melting curves of the PCR process, we observed that the melting curves of osterix and NFATc1 of ADSC‐sEVs were consistent with those of ADSCs, indicating that osterix and NFATc1 mRNA were present in sEVs (Figure S6). Importantly, osterix mRNA in ADSC‐sEVs was highly expressed compared with other mRNAs (Figure 3B).

An increasing number of separation methods and combinations are available for ADSC‐sEV isolation; however, no single optimal separation approach has achieved high recovery and specificity [15]. In finished ADSC‐sEVs, non‐sEV contaminants were inevitably present, including soluble ingredients and other membrane‐structure particles, which might interfere with the roles played by ADSC‐sEVs [16]. Hence, distinguishing between the functional activities of ADSC‐sEVs and non‐ADSC‐sEVs was challenging. We treated ADSCs with 20 μM GW4869 for 24 h, which has been widely used to block the secretion of EVs from various cell types in vitro [17]. Thus, a non‐ADSC‐sEV group was created for functional assays. In addition, the lEV and sEV groups were distinguished by differential centrifugation (Figure 3C).

The expression level of osterix mRNA in ADSC‐sEVs^+^ was 11 times higher than that in ADSC‐sEVs (Figure 3D), suggesting that the increased osterix mRNA resulted from the osteogenic induction medium. mRNA in the cell supernatant exists in two forms: one bound to proteins as a soluble molecule and the other present within vesicles as carriers [18]. Therefore, further elucidation of the localisation of osterix mRNA is required. In this study, GW4869 and differential centrifugation were used to discern the distinct components within the supernatant. The expression of osterix mRNA in ADSC‐sEVs^+^ (GW4869) was significantly lower than that in ADSC‐sEVs^+^ (Figure 3E). In addition, osterix mRNA was highly expressed in sEVs and not in lEVs (Figure 3F). The comprehensive results indicate that the increased osterix mRNA was mainly derived from ADSC‐sEVs^+^ and not from other components.

### 3.4 BMSC Osteoblast Differentiation Induced by ADSC‐sEVs^+^

**FIGURE 4:**
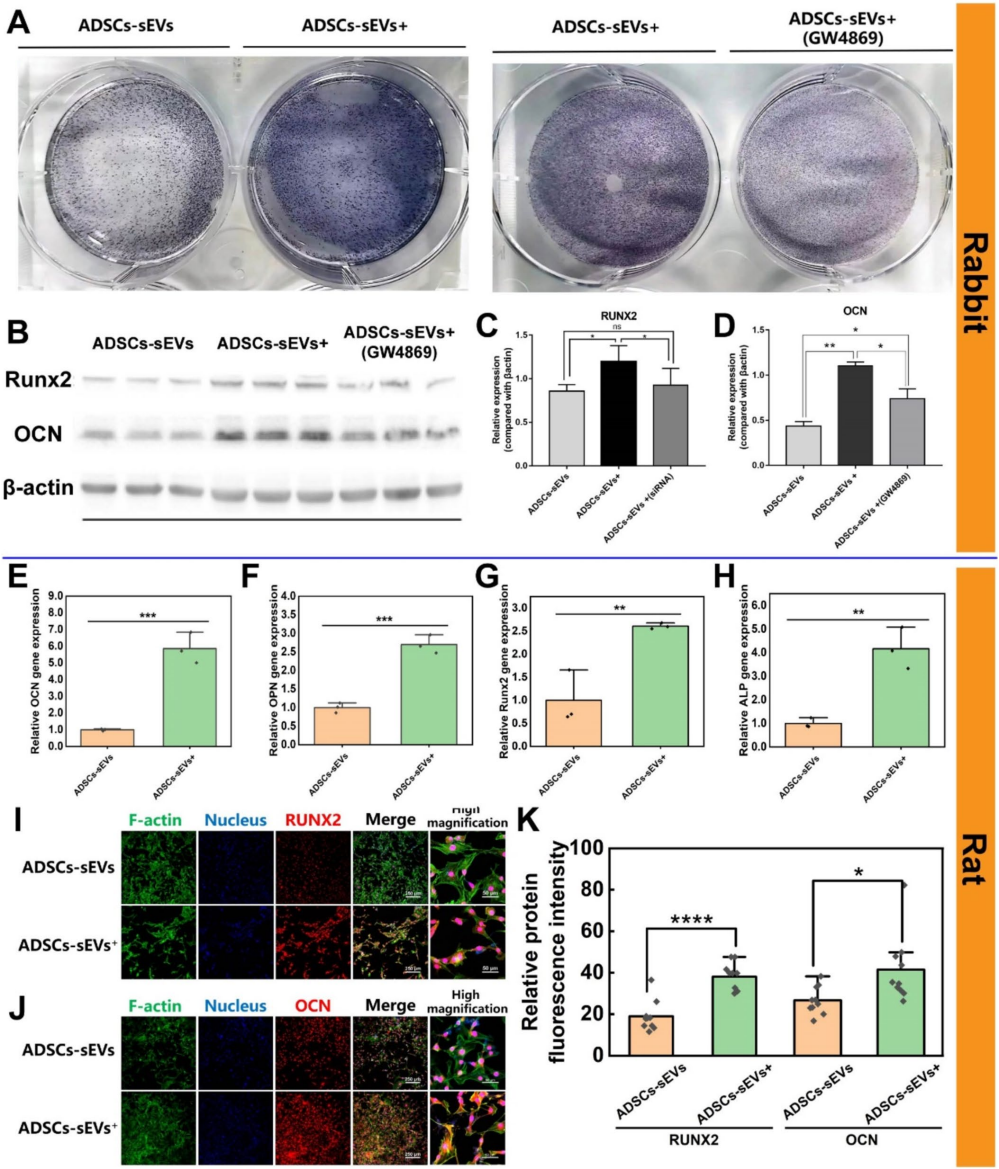
Effects of ADSC‐sEVs^+^ on the osteogenic differentiation of BMSCs. (A) ALP staining of rabbit BMSCs treated with rabbit ADSC‐sEVs, ADSC‐sEVs^+^, and ADSC‐sEVs^+^(GW4869). (B–D) Western blot analysis of osteogenic markers RUNX2 and OCN in rabbit BMSCs treated with rabbit ADSC‐sEVs, ADSC‐sEVs^+^, and ADSC‐sEVs^+^ (GW4869) (*n* = 3, *p < 0.05, **p < 0.01, ns = not significant). (E–H) Expression levels of OCN, OPN, Runx2 and ALP genes in rat BMSCs cultured with ADSC‐sEVs and ADSC‐sEVs^+^ for 5 days (*n* = 3, **p < 0.01, ***p < 0.001). (I, J) Immunofluorescent staining of RUNX2 and OCN in rat BMSCs cultured with rat ADSC‐sEVs and ADSC‐sEVs^+^ for 7 days (scale bars: 250 and 50 μm, respectively). (K) Semi‐quantitative analysis of relative fluorescent intensity per cell of Runx2 and OCN protein via Image J (*n* = 10, *p < 0.05, ****p < 0.0001). ADSCs, adipose mesenchymal stem cells; ALP, alkaline phosphatase; BMSCs, bone marrow stem cells; OCN, osteocalcin; OPN, osteopontin; PBS, phosphate‐buffered saline; RUNX2, runt‐related transcription factor 2; sEVs, small extracellular vesicles.

As detected by ALP staining, rabbit ADSC‐sEVs^+^ promoted osteogenic differentiation of rabbit BMSCs, unlike ADSC‐sEVs (Figure 4A, left). Moreover, rabbit ADSC‐sEV^+^‐triggered osteogenic protein generation was remarkably attenuated in rabbit BMSCs upon intervention with ADSC‐sEVs^+^ (GW4869), as evidenced by a 32%–42% reduction in osteogenic marker expression (Figure 4A–C and Figure S3).

Additionally, after a 5‐day coculture with rat ADSC‐sEVs^+^, the expression of osteogenesis‐related genes in rat BMSCs was significantly upregulated (Figure 4E–H). Immunofluorescence revealed a marked increase in RUNX2 and OCN protein expression in rat BMSCs after a 7‐day coculture with rat ADSC‐sEVs^+^ (Figure 4I–K). Collectively, these data indicate that ADSC‐sEVs, rather than other non‐sEV components, predominantly induce osteoblastic differentiation of BMSCs.

### 3.5 Enhancement of In Vivo Bone Regeneration by ADSC‐sEVs^+^

**FIGURE 5:**
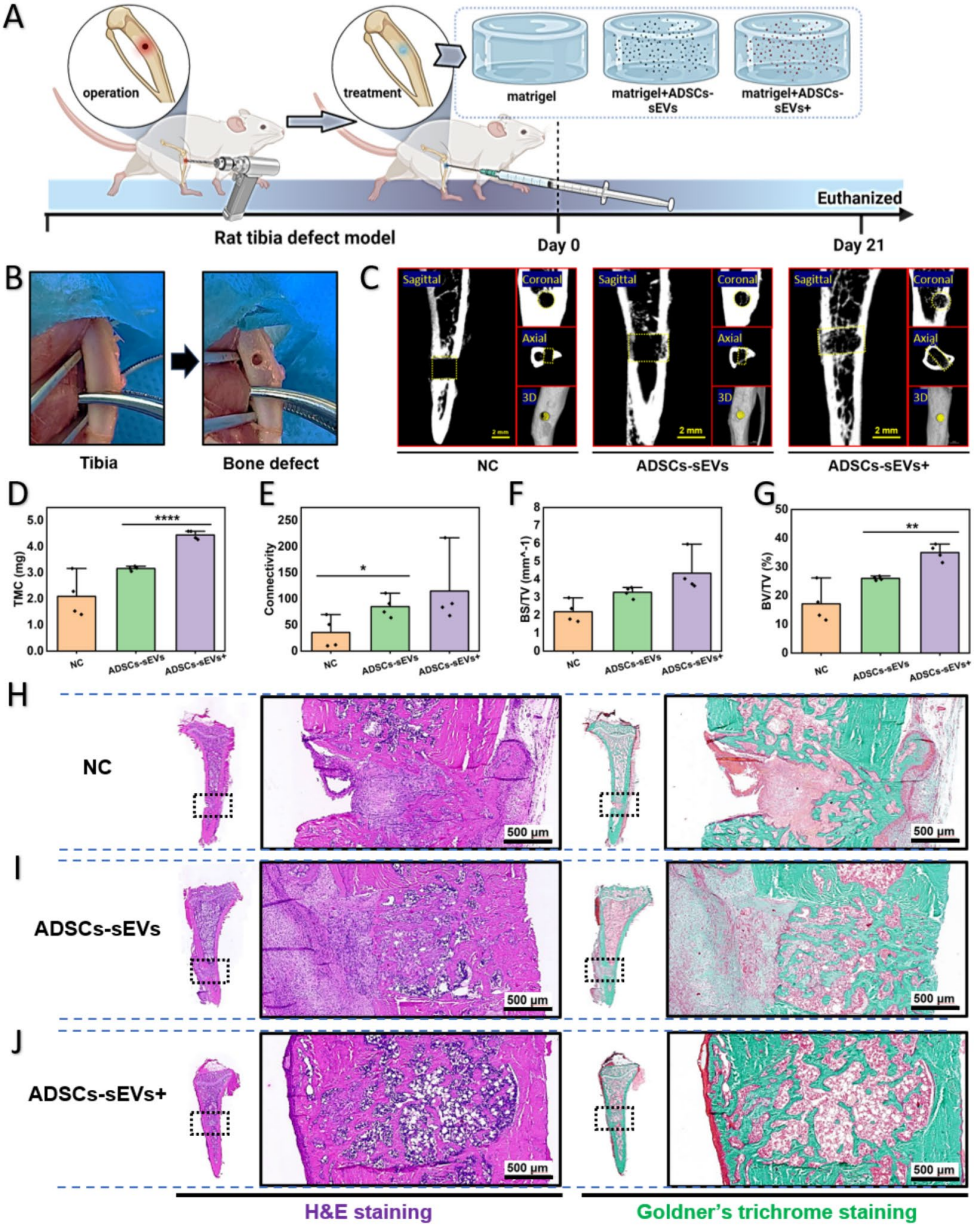
Enhancement of trabecular bone mass by Matrigel‐delivered ADSC‐sEVs^+^. (A) Schematic illustration of the animal experiment. (B) Digital photographs of tibia bone defect modelling in female rats. (C) Representative micro‐CT images of rat tibia defects 3 weeks post‐intervention, including 3D reconstructions and multiplanar reconstructions. Yellow frames in the multiplanar reconstructions indicate bone defect areas; yellow solids in the 3D reconstruction models represent regenerated bone (Scale bar: 2 mm). (D–G) Quantitative micro‐CT analysis of tissue mineral content (TMC), trabecular connectivity, bone surface to tissue volume ratio (BS/TV), and bone volume fraction (BV/TV) at the tibia defect sites of each group (*n* = 4, *p < 0.05, **p < 0.01, ****p < 0.0001). (H–J) Representative images of H&E and Goldner's trichrome staining from negative control (NC), ADSC‐sEVs, and ADSC‐sEVs^+^ groups (Scale bar: 500 μm). ADSCs, adipose mesenchymal stem cells; sEVs, small extracellular vesicles.

A critical‐sized bone defect model was created in the rat tibias to assess the efficacy of ADSC‐sEVs^+^ in promoting bone regeneration in vivo (Figure 5A,B). Three weeks postoperatively, the ADSC‐sEV^+^ group demonstrated increased bone mineralisation and neo‐osteoid formation compared with the NC and ADSC‐sEV groups (Figure 5C). The tissue mineral content (TMC) in the ADSC‐sEV^+^ group was significantly higher than in the ADSC‐sEV group (4.448 ± 0.164 vs. 3.166 ± 0.081, respectively; Figure 5D). With regard to the bone volume fraction (BV/TV) at the site of the bone defect, the ADSC‐sEV^+^ group showed a 1.3‐fold increase compared with the ADSC‐sEV group (0.350 ± 0.028 vs. 0.230 ± 0.007, respectively; Figure 5G). Histological evaluation via HE staining and Goldner's trichrome staining revealed a greater abundance of neo‐osteoids in the ADSC‐sEV^+^ group than in the ADSC‐sEV and NC groups (Figure 5H–J). Taken together, these results indicate that ADSC‐sEVs^+^ enhanced bone regeneration and healing in vivo.

### 3.6 Osterix mRNA Transferred by ADSC‐sEVs^+^ Into BMSCs

**FIGURE 6:**
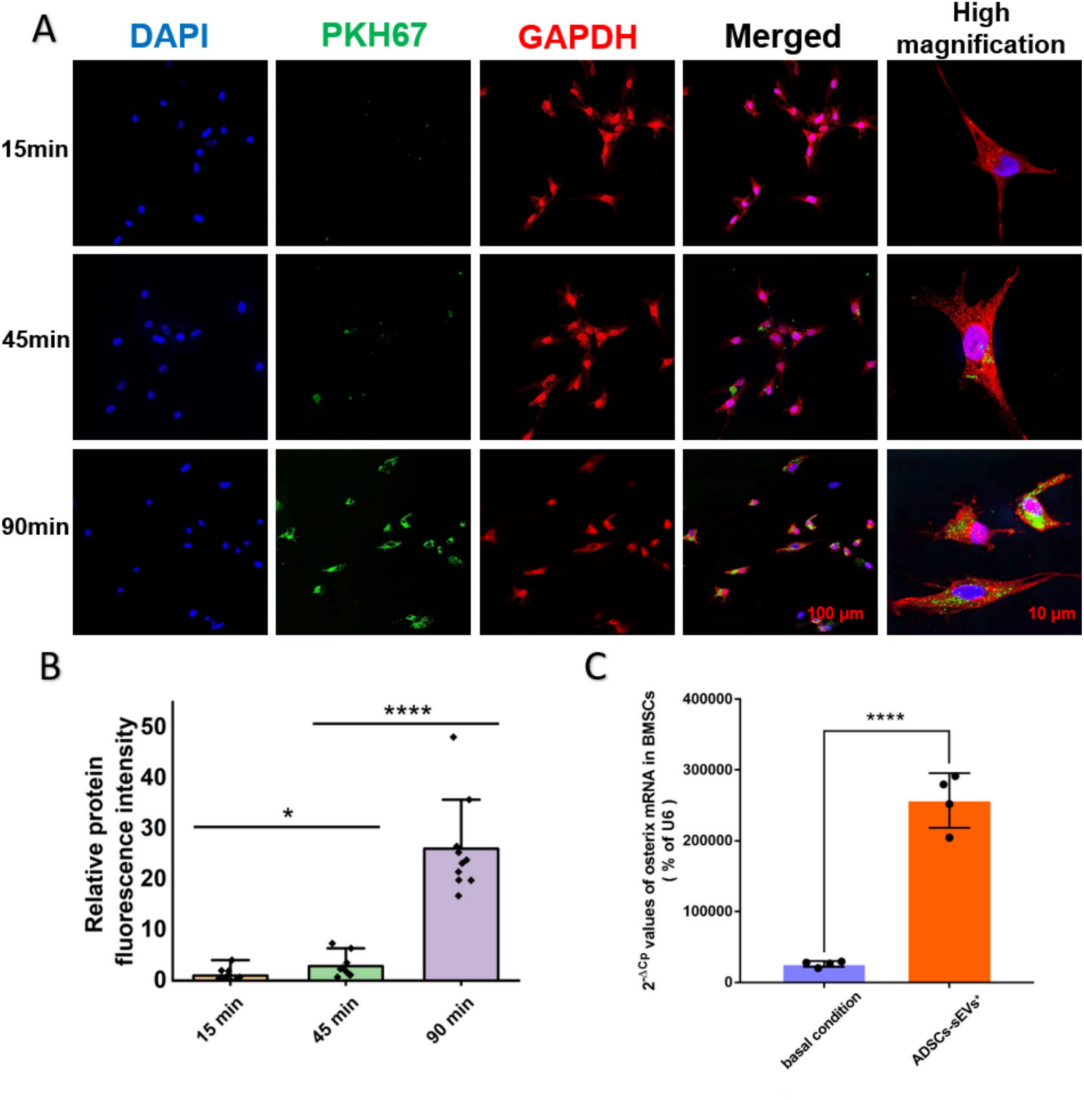
Transport of osterix mRNA into BMSCs by ADSC‐sEVs^+^. (A) Representative immunofluorescence images of BMSCs with ADSC‐sEVs coculture at 15, 45, and 90 min. Green represents ADSC‐sEVs, red labels the cytoskeleton, and blue stains nuclei (Scale bar: 100 and 10 μm, respectively). (B) Semi‐quantitative analysis of relative fluorescent intensity per cell of ADSC‐sEVs via Image J (*n* = 10, *p < 0.05, ****p < 0.0001). (C) Osterix mRNA expression in BMSCs under basal condition and following coculture with ADSC‐sEVs^+^ (*n* = 4, ****p < 0.0001). ADSCs, adipose mesenchymal stem cells; BMSCs, bone marrow stem cells; sEVs, small extracellular vesicles.

Merged images of stained PKH67‐labelled sEVs (green), cytoskeleton (red), and nuclei (blue) revealed the localisation of sEVs in the cytoplasm and around the nuclei (Figure 6A). The green fluorescence intensity of BMSCs at different culture time points indicated that as the coculture period extended, sEVs were progressively internalised by BMSCs (Figure 6B). Compared with BMSCs under basal conditions, BMSCs cocultured with ADSC‐sEVs^+^ showed a significant increase in osterix mRNA, as detected by RT‐PCR (Figure 6C), indicating that osterix mRNA was transferred into BMSCs by ADSC‐sEVs^+^.

### 3.7 Contribution of Osterix mRNA to the Osteogenic Differentiation of BMSCs

**FIGURE 7:**
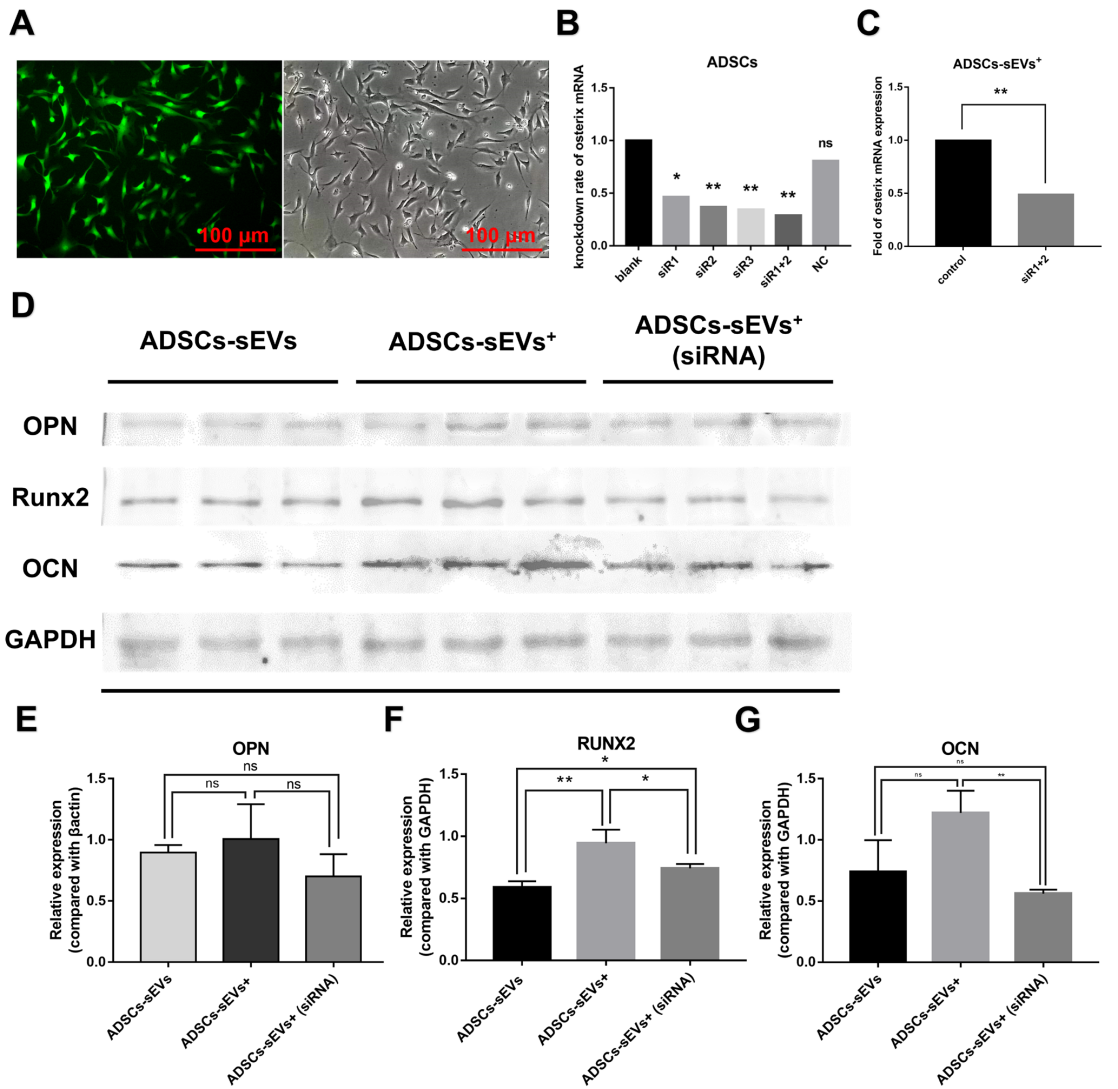
Suppression of osterix mRNA in ADSC‐sEVs^+^ diminishes osteoinductive potential. (A) Representative fluorescence and brightfield microscopy images of ADSCs post‐siRNA transfection (Scale bar: 100 μm). (B) Osterix knockdown efficiency in ADSCs following transfection with distinct siRNA sequences (*n* = 4, *p < 0.05, **p < 0.01, ns = not significant). (C) Relative Osterix mRNA levels in ADSC‐sEVs^+^ (siRNA) normalised to control (*n* = 4, **p < 0.01). (D) Representative western blot images of OPN, RUNX2, and OCN protein expression in BMSCs exposed to ADSC‐sEVs, ADSC‐sEVs^+^, and ADSC‐sEVs^+^ (siRNA). (E–G) The semi‐quantitative assessment of OPN, RUNX2, and OCN protein expression levels following normalisation to loading control (*n* = 3, *p < 0.05, **p < 0.01, ns = not significant). ADSCs, adipose mesenchymal stem cells; BMSCs, bone marrow stem cells; OCN, osteocalcin; OPN, osteopontin; PBS, phosphate‐buffered saline; RUNX2, runt‐related transcription factor 2; sEVs, small extracellular vesicles.

The transfection efficiency of the siRNA‐lipid complex was > 95%, as indicated by the number of fluorescent cells in the fluorescent and general images (Figure 7A). The optimal siRNA for the knockdown of osterix mRNA in ADSCs was the siR1 + 2 combination (Figure 7B). Osterix mRNA expression in ADSC‐sEVs^+^ cells significantly decreased after siR1 + 2 transfection, and ADSC‐sEVs^+^(siRNA) were obtained (Figure 7C). Consequently, the results showed that with the decreased expression of osterix mRNA carried by ADSC‐sEVs^+^, the expression of osteogenic markers on BMSCs, such as OPN, OCN, and RUNX2, was also downregulated (Figure 7D–G and Figure S4).

**FIGURE 8:**
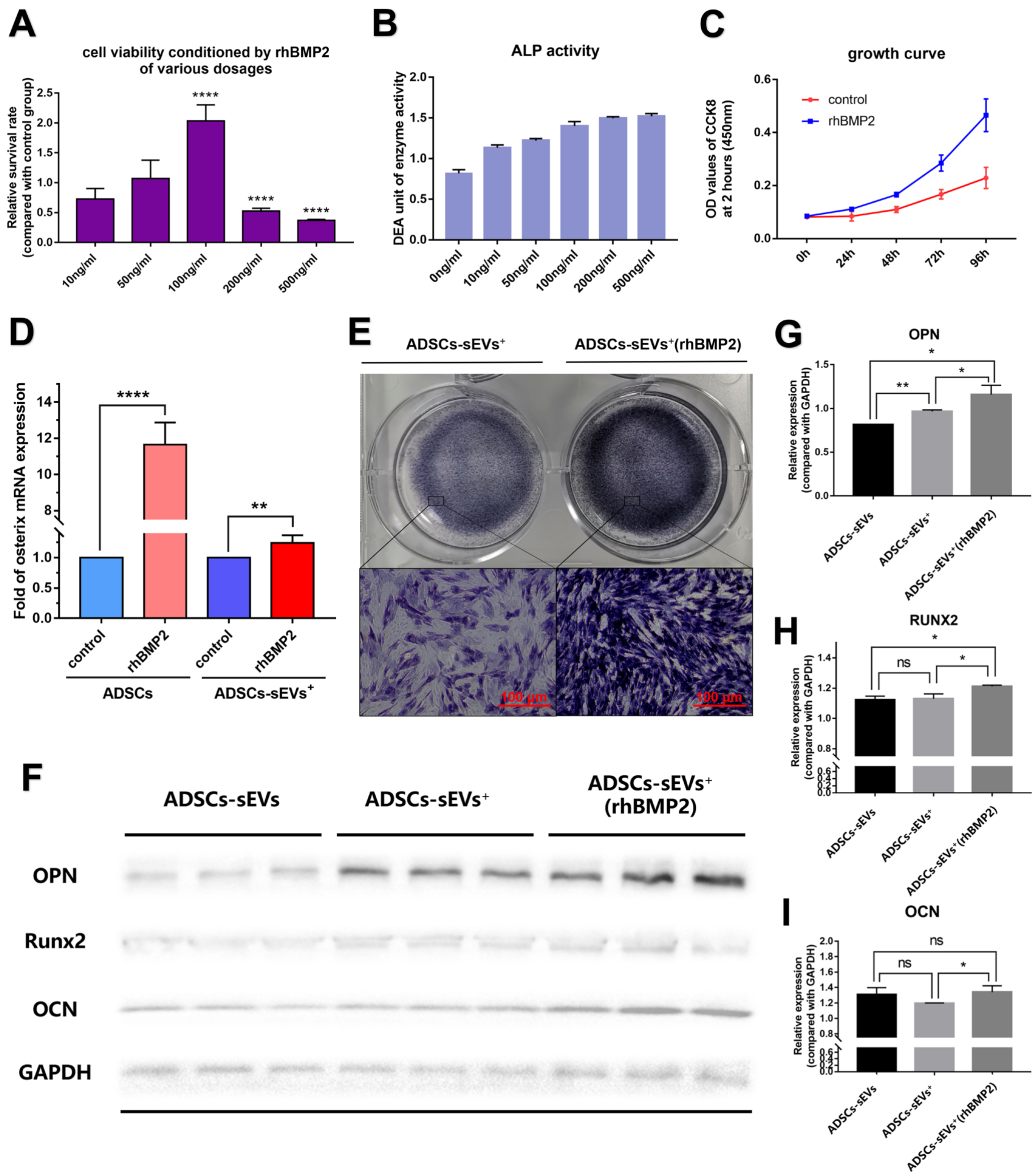
Enhancement of osteoinductive capacity in ADSC‐sEVs^+^ through osterix mRNA upregulation. (A) Assessment of ADSCs viability after treatment with varying concentrations of rhBMP2 using CCK8 assay (*n* = 5, ****p < 0.0001). (B) Osteogenic differentiation of ADSCs evaluated by ALP activity following rhBMP2 stimulation. (C) Growth curves comparing ADSCs with and without 7 days of 100 ng/mL rhBMP2 treatment. (D) Relative expression of Osterix mRNA between ADSCs with and without rhBMP2 stimulation and between ADSC‐sEVs^+^ and ADSC‐sEVs^+^ (rhBMP2) (*n* = 4, **p < 0.01, ****p < 0.0001). (E) ALP staining in BMSCs to compare osteogenic potential between ADSC‐sEVs^+^ and ADSC‐sEVs^+^ (rhBMP2) (Scale bar: 100 μm). (F) Representative western blot images of OPN, RUNX2, and OCN protein expression in BMSCs exposed to ADSC‐sEVs, ADSC‐sEVs^+^, and ADSC‐sEVs^+^ (rhBMP2). (G–I) Semi‐quantitative assessment of OPN, RUNX2, and OCN protein expression levels following normalisation to loading control (*n* = 3, *p < 0.05, **p < 0.01, ns = not significant). ADSCs, adipose mesenchymal stem cells; ALP, alkaline phosphatase; BMSCs, bone marrow stem cells; OCN, osteocalcin; OPN, osteopontin; RUNX2, runt‐related transcription factor 2; sEVs, small extracellular vesicles.

In contrast, a comprehensive assessment of cell viability and osteogenic differentiation induced by various doses of rhBMP2 (Figure 8A,B) revealed that the optimal concentration of rhBMP2 was 100 ng/mL. We successfully isolated ADSC‐sEVs with a high abundance of osterix mRNA and found that the elevated osterix richness in ADSC‐sEVs was attributed to the osterix overexpression in ADSCs stimulated by rhBMP2 for 7 days (Figure 8D). Furthermore, the osteogenic induction effect of ADSC‐sEVs^+^(rhBMP2) was significantly improved by detecting the expression levels of osteogenic proteins in recipient BMSCs (Figure 8F–I and Figure S5) and observing their ALP staining (Figure 8E) compared with the NC group and the ADSC‐sEV^+^ group. Given the critical importance of eliminating residual rhBMP2 from the supernatant to accurately assess the osteogenic potential of ADSC‐sEVs^+^ (rhBMP2), we conducted a concentration analysis of rhBMP2 across various purification steps of ADSC‐sEV^+^ (rhBMP2) samples. Using an ELISA kit, we observed that the concentration of rhBMP2 in sample S4 was nearly seven times lower than that in sample S3. Furthermore, the rhBMP2 concentration in S6 was below the minimum detectable limit of ELISA. These results demonstrate that the combination of ultrafiltration and differential centrifugation effectively removed rhBMP2 from the final product of ADSC‐sEVs^+^ (rhBMP2), thereby eliminating any interference of rhBMP2 in the osteogenic induction of ADSC‐sEVs^+^ (rhBMP2) (Figure S8).

Additionally, the gene expression results of BMSCs in the ADSC‐sEVs^+^(siRNA) and ADSC‐sEVs^+^ (rhBMP2) treatment groups showed the same trend as protein expression (Figure S7). Taken together, osterix mRNA plays a vital role in the osteogenic differentiation of BMSC induced by ADSC‐sEVs^+^.

## 4 Discussion

Despite the immense potential of BMSCs in osteogenic differentiation, their clinical application is limited due to low differentiation efficiency and unclear regulatory mechanisms. Previous studies have shown that ADSC‐sEVs^+^ significantly enhance the osteogenic differentiation of BMSCs, and elucidating the underlying molecular mechanisms may offer novel solutions for addressing clinical application issues associated with BMSCs. Investigating this topic can facilitate the regulation of targeted BMSC differentiation in clinical applications. In the present study, we validated the osteogenic induction effect of ADSC‐sEVs^+^ and explored the underlying mechanisms by which ADSC‐sEVs^+^ induce osteogenic differentiation in BMSCs.

Our study revealed that ADSC‐sEVs^+^ derived from rabbits and rats effectively promoted the osteogenic differentiation of BMSCs. Furthermore, the in vivo study results indicated that ADSC‐sEVs^+^ implantation at the site of bone defects significantly enhanced tissue mineral content and trabecular bone formation, demonstrating the osteoinductive activity of ADSC‐sEVs^+^.

To investigate the changes in composition related to osteogenic induction, we compared the osteogenic mRNA levels within ADSC‐sEVs^+^ and observed a significant increase in the osterix content. Therefore, osterix mRNA in ADSC‐sEVs^+^ may play a crucial role in promoting the osteogenic differentiation of BMSCs.

Osterix is a zinc finger transcription factor belonging to the SP/KIF family [19] that acts downstream of RUNX2/Cbfal during osteoblast differentiation and plays a critical role in osteogenesis and bone tissue formation [20]. RUNX2 has been suggested to be involved in the early stages of osteoblast development, whereas osterix is involved in the maturation stage [21]. In the present study, increased osterix mRNA content caused an increase in RUNX2 protein expression in recipient cells, suggesting that exogenous osterix mRNA is different from the endogenous osterix signalling pathway and is involved in initiating the early stages of osteogenic development. Mice lacking osterix reportedly exhibit impaired bone tissue formation owing to the absence of osteoblasts, resulting in the complete arrest of bone formation [22]. Furthermore, a frameshift mutation in the human osterix gene leads to osteogenesis imperfecta [23]. Therefore, osterix is considered a key regulatory factor in osteoblast differentiation and is involved in regulating the expression of various osteogenic factors. Its mechanism of action involves the regulation of intracellular signalling pathways. However, its potential function as a cell–cell signalling component in the osteogenic differentiation of other cell types remains largely unexplored.

In the domain of extracellular signalling molecules, the qualification of nucleic acids or proteins to fulfil this role faces several formidable barriers. First, they must remain stable in the extracellular environment to evade the ribonuclease clearance mechanisms [24]. Second, they must be taken up by target cells to successfully evade intracellular lysosomal clearance mechanisms [25]. Finally, they regulate the biological processes of target cells [9].

In this study, we confirmed that osterix mRNA was primarily localised to sEVs rather than to lEVs or soluble molecules. sEVs serve as efficient vehicles for long‐distance signal delivery, facilitating cell entry through mechanisms such as membrane fusion, vesicle protein‐mediated endocytosis, and phagocytosis [26]. This natural attribute lays the foundation for the stable persistence of osterix mRNA in the extracellular environment.

Moreover, our immunofluorescence results demonstrated that ADSC‐sEVs^+^ were phagocytosed by BMSCs. RT‐PCR data revealed that the level of osterix mRNA in BMSCs after the uptake of ADSC‐sEVs^+^ was 9.87‐fold higher than the baseline levels. These data suggest that osterix mRNA was taken up by BMSCs and escaped the intracellular clearance mechanisms. Furthermore, functional experiments indicated that a high content of osterix mRNA delivered via ADSC‐sEVs^+^ corresponded to a more pronounced degree of osteogenic differentiation of BMSCs into osteoblasts. This finding suggests that exogenous osterix mRNA delivery has the capacity to regulate the osteogenic differentiation of BMSCs.

**FIGURE 9:**
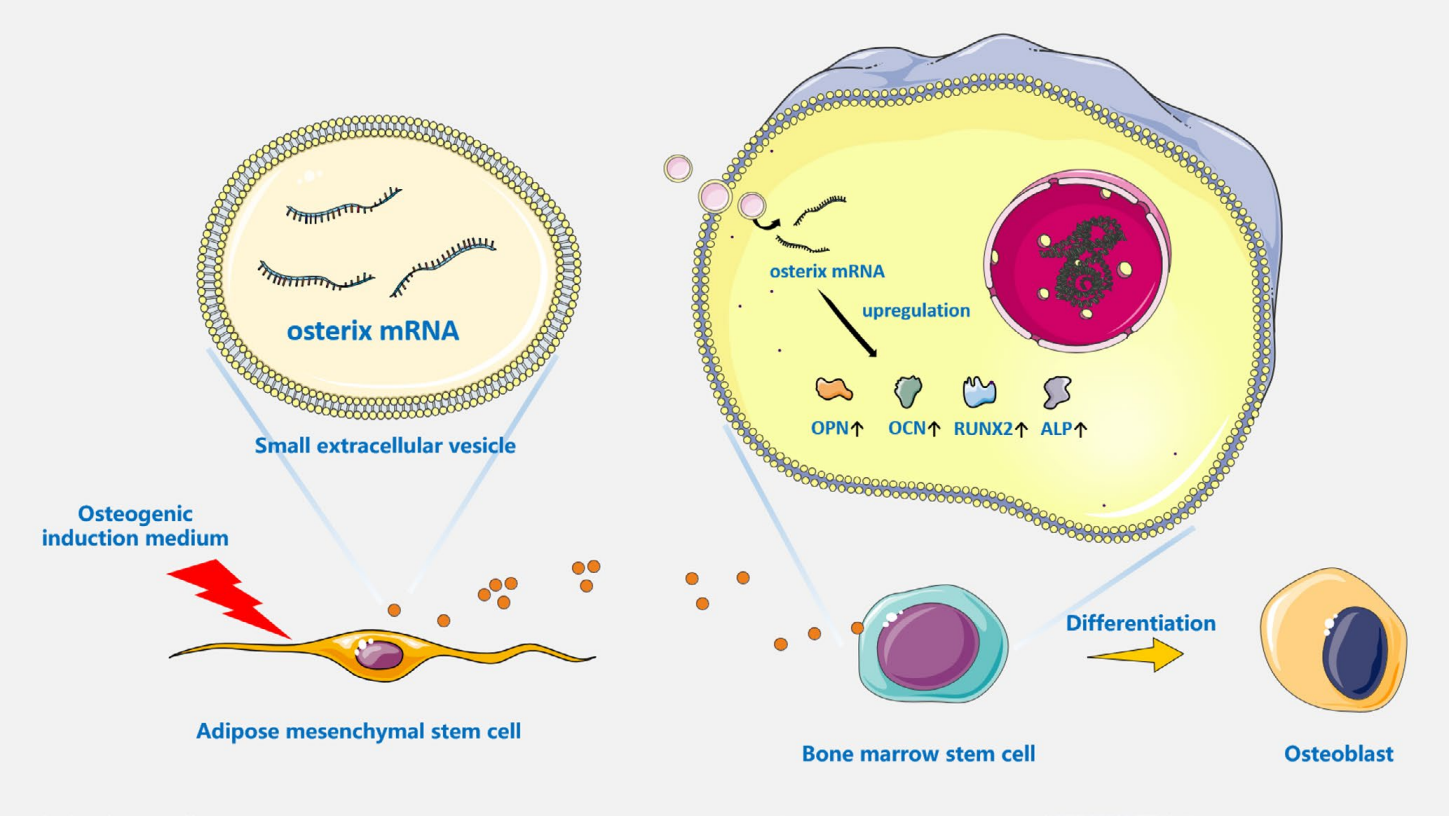
Schematic representation of the mechanism by which ADSC‐sEVs^+^ promote osteogenic differentiation of BMSCs. Upon osteogenic induction, ADSCs release sEVs enriched with osteogenic mRNAs, including osterix. These sEVs facilitate the internalisation of osterix mRNA by BMSCs, thereby upregulating the expression of downstream osteoblast markers and enhancing their differentiation into osteoblasts. ADSCs, adipose mesenchymal stem cells; BMSCs, bone marrow stem cells; sEVs, small extracellular vesicles.

Taken together, our data showed that ADSC‐sEVs^+^ facilitated the differentiation of BMSCs into osteoblasts through the delivery of osterix mRNA (Figure 9), indicating that osterix mRNA can function as a cell–cell signalling molecule participating in remote regulation. This exogenous supplementation of target cellular mRNA may emerge as a novel strategy for enhancing cell‐based therapies.

### 4.1 Limitations of Our Study

First, although we excluded the interference of rhBMP2 in the extraction process of sEVs, measures to indirectly manipulate the content of osterix mRNA in ADSC‐sEVs by upregulating or downregulating the secretion of osterix mRNA in parent cells may still introduce unexpected contaminants. Ideally, the direct genetic engineering of ADSC‐sEVs should be conducted to eliminate other potentially active molecules and eliminate the issue of accidental changes in secretory cells. Second, the integrity of osterix mRNA in ADSC‐sEVs^+^ remains unclear, specifically whether it is intact or partially related to the activation of downstream osteoblast‐related pathways in BMSCs. Future studies could utilise gene sequencing and more in‐depth mechanistic research to determine whether the signalling pathway of exogenous osterix mRNA is identical to that of endogenous osterix in the regulation of cell osteogenic differentiation.

## 5 Conclusions

Our study confirmed the osteogenic activity of ADSC‐sEVs^+^ and identified osterix mRNA in ADSC‐sEVs^+^ as a crucial component in regulating the osteogenic differentiation of target BMSCs. These findings offer valuable insights for the development of innovative approaches to promote the directional differentiation of BMSCs in orthopaedic therapy.

## Author Contributions

**Zhaoquan Liang:** data curation (equal), methodology (lead), project administration (equal), supervision (lead), visualization (lead), writing – original draft (lead). **Yuelin Wu:** data curation (equal), software (equal), writing – original draft (equal). **Junhao Bao:** data curation (equal), formal analysis (equal). **Qiang Xiao:** conceptualization (equal), methodology (equal). **Sidong Luo:** methodology (equal), validation (equal). **Xinfang Liu:** methodology (equal), validation (equal). **Yeyang Wang:** conceptualization (equal), methodology (equal). **Chao Xie:** conceptualization (lead), supervision (lead), writing – review and editing (lead). **Li Zhang:** conceptualization (lead), funding acquisition (lead), supervision (lead).

## Ethics Statement

This study was approved by the Medical Ethics Committee of Guangdong Second Provincial General Hospital. All experimental animal protocols followed the guidelines of the Animal Care and Use Committee of Guangdong Medical Laboratory Animal Centre, Foshan, China [SCXK (Guangdong) 2019‐0035].

## Conflicts of Interest

The authors declare no conflicts of interest.

## Supporting information


**Figure S1.** Flow cytometric characterisation of rat ADSCs and BMSCs. The positive rates for CD29 and CD90 in ADSCs and BMSCs were as high as 95.3% and 77.1% and 99.8% and 99.9%, respectively, whereas CD45 protein expression was negative in both cell types. BMSCs, bone marrow stem cells; ADSCs, adipose mesenchymal stem cells.
**Figure S2.** Western blot analysis of ADSCs lysate (CL), PBS as a negative control (NC), and sEVs for the detection of endosomal protein markers CD63 and TSG101, corresponding to the findings presented in Figure 2D. ADSCs, adipose mesenchymal stem cells; BMSCs, bone marrow stem cells; PBS, phosphate‐buffered saline.
**Figure S3.** Western blot analysis depicting the expression of RUNX2 and OCN in BMSCs following exposure to ADSC‐sEVs, ADSC‐sEVs^+^, and ADSC‐sEVs^+^ (GW4869), corresponding to the findings presented in Figure 4B. ADSCs, adipose mesenchymal stem cells; BMSCs, bone marrow stem cells; OCN, osteocalcin; OPN, osteopontin; RUNX2, runt‐related transcription factor 2; sEVs, small extracellular vesicles.
**Figure S4.** Western blot analysis of OPN, RUNX2, and OCN protein expression in BMSCs following exposure to ADSC‐sEVs, ADSC‐sEVs^+^, and ADSC‐sEVs^+^ (siRNA), which is related to Figure 7D. ADSCs, adipose mesenchymal stem cells; BMSCs, bone marrow stem cells; OCN, osteocalcin; OPN, osteopontin; RUNX2, runt‐related transcription factor 2; sEVs, small extracellular vesicles.
**Figure S5.** Western blot analysis of OPN, RUNX2, and OCN protein expression in BMSCs following exposure to ADSC‐sEVs, ADSC‐sEVs^+^, and ADSC‐sEVs^+^ (rhBMP2), which is related to Figure 8F. ADSCs, adipose mesenchymal stem cells; BMSCs, bone marrow stem cells; OCN, osteocalcin; OPN, osteopontin; RUNX2, runt‐related transcription factor 2; sEVs, small extracellular vesicles.
**Figure S6.** Melting curves of osteogenically related mRNAs during the PCR process. All mRNAs in ADSCs exhibited distinct and singular peak melting curves, whereas osterix and NFATc1 displayed single peak melting curves in ADSC‐sEVs, whereas other mRNAs demonstrated complex multi‐peak melting curves (*n* = 5). ADSCs, adipose mesenchymal stem cells; NFATc1, nuclear factor of activated T‐cells c1; sEVs, small extracellular vesicles.
**Figure S7.** The expression of the osteogenosis‐related genes (RUNX2, OPN, OCN, and ALP) in rabbit BMSCs cocultured with different samples for 7 days (*n* = 3, **p < 0.01, ***p < 0.001, ****p < 0.0001, ns = not significant). ALP, alkaline phosphatase; BMSCs, bone marrow stem cells; OCN, osteocalcin; OPN, osteopontin; RUNX2, runt‐related transcription factor 2.
**Figure S8.** Purification process and rhBMP2 contamination assessment of ADSC‐sEVs^+^ (rhBMP2). (A) Schematic overview of the ADSC‐sEVs purification protocol. (B) ELISA standard curve for rhBMP2, indicating the calibration range and sensitivity of the assay. (C, D) Concentration measurements of residual rhBMP2 in ADSC‐sEVs^+^ (rhBMP2) samples across different preparation stages, as determined by ELISA (*n* = 3, ****p < 0.0001). ADSCs, adipose mesenchymal stem cells; ELISA, enzyme‐linked immunosorbent assay; sEVs, small extracellular vesicles.

## Data Availability

The data that support the findings of this study are available from the corresponding author upon reasonable request.

## References

[1] H. Lin , J. Sohn , H. Shen , M. T. Langhans , and R. S. Tuan , “Bone Marrow Mesenchymal Stem Cells: Aging and Tissue Engineering Applications to Enhance Bone Healing,” Biomaterials 203 (2019): 96–110, 10.1016/j.biomaterials.2018.06.026. 29980291 PMC6733253

[2] C. Cavallo , G. Merli , R. M. Borzì , et al., “Small Extracellular Vesicles From Adipose Derived Stromal Cells Significantly Attenuate In Vitro the NF‐κB Dependent Inflammatory/Catabolic Environment of Osteoarthritis,” Scientific Reports 11, no. 1 (2021): 1053, 10.1038/s41598-020-80032-7. 33441764 PMC7806716

[3] L. Mazini , L. Rochette , M. Amine , and G. Malka , “Regenerative Capacity of Adipose Derived Stem Cells (ADSCs), Comparison With Mesenchymal Stem Cells (MSCs),” International Journal of Molecular Sciences 20, no. 10 (2019): 2523, 10.3390/ijms20102523. 31121953 PMC6566837

[4] S. R. Baglio , K. Rooijers , D. Koppers‐Lalic , et al., “Human Bone Marrow‐ and Adipose‐Mesenchymal Stem Cells Secrete Exosomes Enriched in Distinctive miRNA and tRNA Species,” Stem Cell Research & Therapy 6 (2015): 127, 10.1186/s13287-015-0116-z. 26129847 PMC4529699

[5] K. S. Lee , J. Lee , H. K. Kim , et al., “Extracellular Vesicles From Adipose Tissue‐Derived Stem Cells Alleviate Osteoporosis Through Osteoprotegerin and miR‐21‐5p,” Journal of Extracellular Vesicles 10, no. 12 (2021): e12152, 10.1002/jev2.12152. 34596354 PMC8485335

[6] C. Gu , H. Zhang , and Y. Gao , “Adipose Mesenchymal Stem Cells‐Secreted Extracellular Vesicles Containing microRNA‐192 Delays Diabetic Retinopathy by Targeting ITGA1,” Journal of Cellular Physiology 236, no. 7 (2021): 5036–5051, 10.1002/jcp.30213. 33325098

[7] Y. Song , H. Li , X. Ren , H. Li , and C. Feng , “SNHG9, Delivered by Adipocyte‐Derived Exosomes, Alleviates Inflammation and Apoptosis of Endothelial Cells Through Suppressing TRADD Expression,” European Journal of Pharmacology 872 (2020): 172977, 10.1016/j.ejphar.2020.172977. 32007500

[8] W. Li , Y. Liu , P. Zhang , et al., “Tissue‐Engineered Bone Immobilized With Human Adipose Stem Cells‐Derived Exosomes Promotes Bone Regeneration,” ACS Applied Materials & Interfaces 10, no. 9 (2018): 5240–5254, 10.1021/acsami.7b17620. 29359912

[9] K. O'Brien , K. Breyne , S. Ughetto , L. C. Laurent , and X. O. Breakefield , “RNA Delivery by Extracellular Vesicles in Mammalian Cells and Its Applications,” Nature Reviews Molecular Cell Biology 21, no. 10 (2020): 585–606, 10.1038/s41580-020-0251-y. 32457507 PMC7249041

[10] D. S. Amarasekara , S. Kim , and J. Rho , “Regulation of Osteoblast Differentiation by Cytokine Networks,” International Journal of Molecular Sciences 22, no. 6 (2021): 2851, 10.3390/ijms22062851. 33799644 PMC7998677

[11] Y. Wang , C. Li , R. Zhao , et al., “CircUbe3a From M2 Macrophage‐Derived Small Extracellular Vesicles Mediates Myocardial Fibrosis After Acute Myocardial Infarction,” Theranostics 11, no. 13 (2021): 6315–6333, 10.7150/thno.52843. 33995660 PMC8120198

[12] K. Rajagopal and V. Madhuri , “Comparing the Chondrogenic Potential of Rabbit Mesenchymal Stem Cells Derived From the Infrapatellar Fat Pad, Periosteum & Bone Marrow,” Indian Journal of Medical Research 154, no. 5 (2021): 732–742, 10.4103/ijmr.IJMR_93_19. 35532591 PMC9210523

[13] T. C. Lee , T. H. Lee , Y. H. Huang , et al., “Comparison of Surface Markers Between Human and Rabbit Mesenchymal Stem Cells,” PLoS One 9, no. 11 (2014): e111390, 10.1371/journal.pone.0111390. 25380245 PMC4224397

[14] S. Lapi , F. Nocchi , R. Lamanna , et al., “Different Media and Supplements Modulate the Clonogenic and Expansion Properties of Rabbit Bone Marrow Mesenchymal Stem Cells,” BMC Research Notes 1 (2008): 53, 10.1186/1756-0500-1-53. 18710506 PMC2525639

[15] C. Théry , K. W. Witwer , E. Aikawa , et al., “Minimal Information for Studies of Extracellular Vesicles 2018 (MISEV2018): A Position Statement of the International Society for Extracellular Vesicles and Update of the MISEV2014 Guidelines,” Journal of Extracellular Vesicles 7 (2018): 1535750, 10.1080/20013078.2018.1535750. 30637094 PMC6322352

[16] R. Poupardin , M. Wolf , and D. Strunk , “Adherence to Minimal Experimental Requirements for Defining Extracellular Vesicles and Their Functions,” Advanced Drug Delivery Reviews 176 (2021): 113872, 10.1016/j.addr.2021.113872. 34284058

[17] M. Catalano and L. O'Driscoll , “Inhibiting Extracellular Vesicles Formation and Release: A Review of EV Inhibitors,” Journal of Extracellular Vesicles 9 (2020): 1703244, 10.1080/20013078.2019.1703244. 32002167 PMC6968539

[18] X. Chen , H. Liang , J. Zhang , K. Zen , and C. Y. Zhang , “Secreted microRNAs: A New Form of Intercellular Communication,” Trends in Cell Biology 22, no. 3 (2012): 125–132, 10.1016/j.tcb.2011.12.001. 22260888

[19] L. A. Kaback , Y. Soung , A. Naik , et al., “Osterix/Sp7 Regulates Mesenchymal Stem Cell Mediated Endochondral Ossification,” Journal of Cellular Physiology 214, no. 1 (2008): 173–182, 10.1002/jcp.21176. 17579353

[20] L. Zou , X. Zou , H. Li , et al., “Molecular Mechanism of Osteochondroprogenitor Fate Determination During Bone Formation,” Advances in Experimental Medicine and Biology 585 (2006): 431–441, 10.1007/978-0-387-34133-0_28. 17120800

[21] T. Komori , “Regulation of Bone Development and Maintenance by Runx2,” Frontiers in Bioscience 13 (2008): 898–903, 10.2741/2730. 17981598

[22] X. Zhou , Z. Zhang , J. Q. Feng , et al., “Multiple Functions of Osterix Are Required for Bone Growth and Homeostasis in Postnatal Mice,” Proceedings of the National Academy of Sciences of the United States of America 107, no. 29 (2010): 12919–12924, 10.1073/pnas.0912855107. 20615976 PMC2919908

[23] P. Lapunzina , M. Aglan , S. Temtamy , et al., “Identification of a Frameshift Mutation in Osterix in a Patient With Recessive Osteogenesis Imperfecta,” American Journal of Human Genetics 87, no. 1 (2010): 110–114, 10.1016/j.ajhg.2010.05.016. 20579626 PMC2896769

[24] J. P. Tosar , K. Witwer , and A. Cayota , “Revisiting Extracellular RNA Release, Processing, and Function,” Trends in Biochemical Sciences 46, no. 6 (2021): 438–445, 10.1016/j.tibs.2020.12.008. 33413996 PMC8122015

[25] J. Gilleron , W. Querbes , A. Zeigerer , et al., “Image‐Based Analysis of Lipid Nanoparticle‐Mediated siRNA Delivery, Intracellular Trafficking and Endosomal Escape,” Nature Biotechnology 31, no. 7 (2013): 638–646, 10.1038/nbt.2612. 23792630

[26] A. Turchinovich , O. Drapkina , and A. Tonevitsky , “Transcriptome of Extracellular Vesicles: State‐of‐the‐Art,” Frontiers in Immunology 10 (2019): 202, 10.3389/fimmu.2019.00202. 30873152 PMC6404625

