# Supplementary material for: Osterix mRNA Enrichment in Small Extracellular Vesicles Derived From Osteogenically Induced ADSCs: A Promoter of Osteogenic Differentiation in BMSCs
Source: J Cell Mol Med. 2025 Jan 13;29(1):e70353. doi: 10.1111/jcmm.70353 (PMC11727376; doi:10.1111/jcmm.70353)
Supplement: Supplementary file 1 — Figure S1. Flow cytometric characterisation of rat ADSCs and BMSCs. The positive rates for CD29 and CD90 in ADSCs and BMSCs were as high as 95.3% and 77.1% and 99.8% and 99.9%, respectively, whereas CD45 protein expression was negative in both cell types. BMSCs, bone marrow stem cells; ADSCs, adipose mesenchymal stem cells. Figure S2. Western blot analysis of ADSCs lysate (CL), PBS as a negative control (NC), and sEVs for the detection of endosomal protein markers CD63 and TSG101, corresponding to the findings presented in Figure 2D. ADSCs, adipose mesenchymal stem cells; BMSCs, bone marrow stem cells; PBS, phosphate‐buffered saline. Figure S3. Western blot analysis depicting the expression of RUNX2 and OCN in BMSCs following exposure to ADSC‐sEVs, ADSC‐sEVs+, and ADSC‐sEVs+ (GW4869), corresponding to the findings presented in Figure 4B. ADSCs, adipose mesenchymal stem cells; BMSCs, bone marrow stem cells; OCN, osteocalcin; OPN, osteopontin; RUNX2, runt‐related transcription factor 2; sEVs, small extracellular vesicles. Figure S4. Western blot analysis of OPN, RUNX2, and OCN protein expression in BMSCs following exposure to ADSC‐sEVs, ADSC‐sEVs+, and ADSC‐sEVs+ (siRNA), which is related to Figure 7D. ADSCs, adipose mesenchymal stem cells; BMSCs, bone marrow stem cells; OCN, osteocalcin; OPN, osteopontin; RUNX2, runt‐related transcription factor 2; sEVs, small extracellular vesicles. Figure S5. Western blot analysis of OPN, RUNX2, and OCN protein expression in BMSCs following exposure to ADSC‐sEVs, ADSC‐sEVs+, and ADSC‐sEVs+ (rhBMP2), which is related to Figure 8F. ADSCs, adipose mesenchymal stem cells; BMSCs, bone marrow stem cells; OCN, osteocalcin; OPN, osteopontin; RUNX2, runt‐related transcription factor 2; sEVs, small extracellular vesicles. Figure S6. Melting curves of osteogenically related mRNAs during the PCR process. All mRNAs in ADSCs exhibited distinct and singular peak melting curves, whereas osterix and NFATc1 displayed single peak melting curves in AD [file JCMM-29-e70353-s001.docx]

**Supplementary information**

**Osterix mRNA Enrichment in Small Extracellular Vesicles Derived from Osteogenically Induced ADSCs: A Promoter of Osteogenic Differentiation in BMSCs**

Zhaoquan Liang^1†^, Yuelin Wu^1†^, Junhao Bao^1^, Qiang Xiao^1^, Sidong Luo^1^, Xinfang Liu^1^, Yeyang Wang^1^, Chao Xie^2^* and Li Zhang^3^*

1. Department of Spine, Orthopaedic Center, Guangdong Second Provincial General Hospital, Southern Medical University, Guangzhou, Guangdong, P. R. China.

2. Department of Joint and Orthopedics, Zhujiang Hospital, Southern Medical University, Guangzhou, Guangdong, P. R. China.

3. Department of Spine, Orthopaedic Center, Guangdong Second Provincial General Hospital, Jinan University, Guangzhou, Guangdong, P. R. China.

^†^ These authors contributed equally to this work.

* **Correspondence**

Chao Xie, Department of Joint and Orthopedics, Zhujiang Hospital, Southern Medical University, Guangzhou, Guangdong, P. R. China.

Email: chaoxie1118@foxmail.com

Li Zhang, Department of Spine, Orthopaedic Center, Guangdong Second Provincial General Hospital, Jinan University, Guangzhou, Guangdong, P. R. China.

Email: [Lizhang686@163.com](mailto:Lizhang686@163.com)

1. **Supplementary Materials and Methods**

**1.1 ADSCs and BMSCs harvesting**

Male New Zealand white rabbits aged 2-3 months (purchased from Guangdong Medical Laboratory Animal Center) and female SD rats aged 5-6 months (purchased from Zhuhai Bestest Biotech Co., Ltd.) were kept in the Animal Center of Guangdong Second Provincial General Hospital. All experimental operations complied with the relevant requirements of the Ethical Committee of Guangdong Second Provincial General Hospital to minimize the pain of experimental animals. In order to meet the "3R principle" and "5F" of animal welfare, all rabbits were quarantined for three days, housed in a single cage, fed at least twice a day, and kept in a clean and dry environment. In the process of feeding, once the rabbit suffered from unexpected pain, weight loss, loss of appetite, weakness, and other conditions, the experiment was immediately terminated, and the rabbit was euthanized. Experimental Procedures of executing rabbits to provide subcutaneous groin adipose tissue and femur-knee-tibias for MSCs extraction are as followed.

**1.1.1 Removal of the tibia, fibula, and fat**

All surgical instruments were sterilized by high-pressure steam before use. Rabbits and rats were executed with an overdose of pentobarbital, the operating area (groin and hind legs) was shaved, and the skin of the operating area was sterilized with iodophor three times. Then disposable sterile surgical drapes were placed on the operating area. Then the skin was cut to expose the subcutaneous inguinal fat, bilateral tibia, and fibula. The fat, tibia, and fibula were cut off and placed in cell culture dishes containing antibacterial (400 U/mL) phosphate-buffered saline (PBS), respectively.

**1.1.2 Digestion and centrifugation of tissue cells**

Type I collagenase solution (3 mg/mL) was prepared according to the product instructions. The type I collagenase was added to the cut (1mm³) tissue and digested at 37 °C incubator for 60 minutes. After that, the tissue block was filtered with a 40-um filter membrane, and then the filter membrane was washed with 5 mL PBS. The filtrate was collected in a 50 mL centrifuge tube and placed in a centrifuge at 1200 rpm for 10 minutes. Then, the supernatant was removed, and the precipitate was resuspended with PBS. The supernatant was centrifuged at 1200 rpm for 10 minutes, then removed, and the precipitate was resuspended with complete culture and inoculated into a 25 cm² culture flask.

**1.1.3 ADSCs and BMSCs extraction**

The blood vessels and lymph nodes were removed as much as possible before the adipose tissue was cut into approximately 2 mm^3^ small pieces, then soaked in 3 mg/mL collagenase I (Sigma-Aldrich, USA) solution at 37 °C for digestion for 1 hour, and shaken every 20 minutes. After digestion, the mixture was filtered through a 40 μm filter membrane, and the filtrate was centrifuged at 1200 rpm for 10 minutes to remove the supernatant. The precipitate was resuspended by PBS and centrifuged again.

During BMSCs extraction, the epiphyses of the femur and tibia were cut off under sterile conditions, and the bone marrow cavity was washed with Dulbecco's Modified Eagle's Medium (DMEM)/F-12 (1:1) (Thermo Fisher Scientific, USA) using a 2 mL syringe until the bones turned white. The rinse solution was collected and centrifuged at 1200 rpm for 10 minutes. Then, we got cells precipitated on the bottom of the centrifuge tube.

**1.1.4 Cell culture**

After DMEM supplemented with 20% fetal bovine serum (Gibco, USA), 2% penicillin, and streptomycin (Thermo Fisher Scientific, USA) was used to suspend both ADSCs and BMSCs sediments respectively, they were seeded in culture flasks at 37 °C in a humidified incubator containing 5% CO2 for 48 hours. The culture medium was replaced to remove non-adherent cells, and culturing was continued until 80% fusion was achieved. All ADSCs and BMSCs used in the subsequent experiments were at 2-4 passages.

**1.2 Flow cytometry analysis of ADSCs and BMSCs**

To characterize the MSC surface markers of isolated cells, ADSCs and BMSCs at passage 2 were collected and washed twice with ice-cold washing buffer (PBS with 0.5% BSA) by centrifugation at 300 × g for 5 minutes. After the cell concentration was determined by cell count, approximately 10^5^ cells were added to each 1.5 mL Eppendorf tube and blocked with PBS containing 2% BSA on ice for 40 minutes. After two more washes with washing buffer, the cells were centrifuged at 300 × g for 5 minutes and incubated with primary antibodies against CD44 (GeneTex, USA), CD90 (Thermo Fisher, USA), CD29 (Thermo Fisher, USA), CD105 (BD Biosciences, USA), CD34 (GeneTex, USA), and CD45 (GeneTex, USA) for 90 minutes on ice. Then, secondary goat anti-mouse FITC (ABclonal, China) antibodies or goat anti-rat-FITC (ABclonal, China) antibodies were incubated for 60 minutes in the absence of light after two washes of the cells conjugated with primary antibodies. Negative control staining was performed using a non-specific mouse or rat IgG secondary antibody. Subsequently, the cells were fixed with 4% paraformaldehyde (Biosharp, China) and analyzed using a flow cytometer (BD FACSAria™ III, USA) as soon as possible. The resulting graphs were evaluated using the BD FACSDiva™ software (BD Biosciences, USA).

**1.3 ALP staining and ARS staining**

A BCIP/NBT alkaline phosphatase color development kit (Meilunbio, China) was utilized. The working solution, consisting of 3 mL AP reaction buffer per well in six-well plates mixed with 20 μL NBT solution and 30 μL BCIP solution, was prepared in advance. After washing with PBS three times, cells in six-well plates were fixed with 4% paraformaldehyde at room temperature for 30 minutes. Next, Tris-buffered saline tween-20 (TBST) was used to wash the plate three times, followed by a chromogenic reaction through incubation with the working solution for 24 hours at room temperature in a dark room. The cells were washed with PBS, and images were observed using a general microscope.

ARS staining solution (Solar Life Sciences, China) was used to disseminate the cells for 10 minutes after fixation with 4% paraformaldehyde. Subsequently, mineralized nodules were observed after rinsing three times with double-distilled water to remove the residual dye solution. All the washing steps mentioned above should be performed as gently as possible to minimize membrane breakage.

**1.4 Alkaline phosphatase activity analysis**

An alkaline phosphatase assay kit (Beyotime, China) was used to detect ALP activity quantitatively, according to the manufacturer's instructions. Briefly, quantitative chromogenic substrate solution and 0.5 mM P-nitrophenol solution as standard working solutions for reference were prepared on ice, and test samples were prepared by collecting the supernatant of lysed cell suspensions after centrifugation at 10,000 rpm for one minute at four °C and lysis process of the phosphatase inhibitor-free lysate. Test samples, a detection buffer as a blank control, and a standard working solution of gradient concentration were added to the 96-well plate. The corresponding chromogenic substrate solution was then added and incubated at 37 °C for 10 minutes. Next, 100 μL of reaction stop solution was added to each well, and different degrees of yellow reflecting ALP activity was observed. An automatic ELISA analyzer (BioTek ELX800, USA) was used to detect and analyze the optical density of each well at a wavelength of 405 nm.

**1.5 Isolation and Characteristics of ADSC-sEVs**

All the cells used for sEVs isolation were seeded in T75 cell culture flasks. When the cells reached approximately 90% confluence, the culture supernatant was collected 48 hours after the addition of cell-conditioned medium, which consisted of DMEM/F12, 2% penicillin and streptomycin, and 10% serum-free FBS (Biological Industries, Israel). The plate-bottom adherent cells were digested with trypsin for cell counting, and the survival rate was determined by trypan blue staining. Extracellular vesicles were isolated from the collected medium by differential ultracentrifugation. All centrifugation steps were performed at 4 °C. The supernatants were first centrifuged at 2,000 × g for 10 minutes to remove the cells. Next, the supernatant was filtered through a 0.22 μm filter membrane to remove debris and dead cells from the filter residue. The filtrate was further ultra-filtered by centrifugation at 3,200 × g for 10 minutes to enrich EVs, followed by ultracentrifugation at 20,000 × g for 30 minutes using an SW41TI rotor (Optima XPN-100 Ultracentrifuge, Beckman Coulter, USA) to remove LEVs or other membranous structures larger than 200 nm. The supernatant was ultracentrifuged once again at 100,000 × g for 70 minutes, and the pelleted sEVs were resuspended in 200 μL PBS and transferred to a sterile tube, the morphology of isolated sEVs was immediately visualized by transmission electron microscope (TEM; Hitachi, HT-7700, Japan), and the distribution of size was analyzed by nanoparticle tracking analysis (NTA; PARTICLE METRIX, Zeta VIEW, Germany). Immunoblotting was performed to detect the expression of the known sEVs marker CD63. Next, the protein concentrations of the sEVs were measured using an enhanced BCA protein assay kit. ADSC-sEVs diluted in a culture medium were passed through a 0.22 μm filter to be sterilized before subsequent experiments started. Samples were stored at -80 °C for EVs characterization and RNA extraction or -20 °C for protein analysis.

**1.6 TEM and NTA**

Purified ADSC-sEVs were visualized by TEM (Hitachi, HT-7700, Japan). The 10 μL ADSC-sEVs sample was dropped onto the copper wire for precipitation for 1 minute, and the floating liquid was absorbed by filter paper. 10 μL of uranium dioxyacetate was added to the copper wire for precipitation for 1 minute, and the floating solution was absorbed by filter paper. After drying for several minutes at room temperature, electron microscopy was performed at 100 kV.

The frozen samples were removed, thawed in a 25 °C water bath, and placed on ice. ADSC-sEVs samples were diluted with 1×PBS and directly used for the detection of the nanoparticle tracking analyzer (PARTICLE METRIX, ZetaVIEW, Germany), according to the manufacturer's instructions.

**1.7 Western blot analysis**

Western blot was performed on the protein lysates. Briefly, samples of EVs or cells were lysed at 4 °C for 15 minutes in RIPA lysis buffer (Beyotime, China) supplemented with 1% PMSF (Beyotime, China) composed of 1% protease inhibitor cocktail, 1% phosphatase inhibitor cocktail, and 1% 0.25M EDTA (Beyotime, China). The lysate was sonicated three times for 10 seconds on ice to disrupt the membranous structures and extract transmembrane proteins. Protein concentration was measured using an enhanced BCA assay kit (Beyotime, China), and samples were loaded on a polyacrylamide 10% precast gel (Beyotime, China) at a concentration of 30 µg/well and separated by SDS-PAGE. Proteins were transferred onto PVDF membranes using a Trans-Blot Turbo Transfer system (Bio-Rad, Hercules, California, USA). The membranes were immunoblotted with primary antibodies (Table S1) and then incubated with the HRP-conjugated secondary antibody for 1 hour at room temperature. Protein bands were detected and analyzed using an Alliance Q9 Micro Light chemiluminescence imaging system (UVItec Alliance, UK). Images were processed using the open-access software ImageJ (NIH, USA). β-actin and GAPDH were used as an internal control.

**1.8 siRNA transfection of ADSCs**

The siRNA provided by Guangzhou RiboBio Co., Ltd. was used to knock down Osterix mRNA content in ADSC-sEVs. ADSCs were cultured in six-well plates at a concentration of 2 × 10^5^ cells/well and then co-incubated with several different kinds of siRNA (2.5 μg) for approximately 24 or 48 hours. siRNAs designed for knockdown of Osterix mRNA expression were transfected with Lipofectamine 3000 reagent (Life Technologies Invitrogen, USA). Transfection efficiency was analyzed by observing fluorescence expression using an inverted fluorescence microscope (Leica DMIL LED, Germany). After determining the optimal siRNA-lipid complex to maximize transfection efficiency, lysates and the corresponding medium were used to analyze the expression of Osterix mRNA in subsequent studies.

**1.9 Cellular uptake assay and immunofluorescence**

To monitor ADSC-sEVs uptake, the PKH67 Fluorescent Cell Linker Kit (Sigma Aldrich, USA) was used to label the membranes of the ADSC-sEVs according to the manufacturer's instructions. In detail, 0.8 μL of PKH67 (1 mM) diluted in 100 μL of dilution C was prepared at 37 °C after an ultrasonic bath for 15 minutes, and 20 μg of ADSC-sEVs was diluted in another 100 μL of dilution C. Then, the diluted PKH67 and ADSC-sEVs were mixed and incubated for one hour in the dark. The mixture diluted in 10 mL PBS was added to 2 mL of sucrose (20%) in a tube for ultracentrifugation at 100,000 × g for 70 minutes at 4 °C, which helped to avoid the interference of unbound PKH67 in the follow-up experiment. The labeled pellets were resuspended in 50 μL PBS.

BMSCs were seeded onto 22 mm diameter poly L-lysine-coated coverslips. After adherence, the cells were maintained in DMEM/F-12 containing 10% fetal bovine serum, 5 U/mL penicillin, 5 μg/mL streptomycin, and the fluorescently labeled ADSC-sEVs of 50 μL at 37 °C in a 95% air/5% CO2 atmosphere. After co-incubation with ADSC-sEVs labeled with green fluorescence for 15, 45, and 90 minutes, the medium was removed, followed by three rinses for 10 minutes each time, and the cells were fixed with 4% paraformaldehyde at room temperature for 20 minutes. TritonX-100 (Sigma Aldrich, USA) was added for 30 minutes to rupture in membrane. 3 μL of rabbit anti-GAPDH (Thermo Fisher, USA) was diluted with 297 μL of PBS (final concentration 1:100) and pipetted into the center of the dishes overnight. The next day, the primary antibody was recycled, and goat anti-rabbit IgG (H+L) Cross-Adsorbed ReadyProbes™ secondary antibody (Thermo Fisher, USA) was used to stain the cells for 30 minutes. After three washes with PBS, DAPI (Sigma Aldrich, USA) was used to stain the nuclei blue, and all samples were photographed under a confocal light microscope (STELLARIS 5 Cryo, Leica, Germany).

**2.0 ELISA**

To demonstrate potential contamination of SEV by rhBMP2, we assayed samples (S1, S2, S3, S4, S5, S6) obtained from various preparation steps using the manufacturer's instructions for the rhBMP-2 ELISA kit (Ruida Henghui, China). Based on the rhBMP2 standard curve, the fitting function was derived as $C=\sqrt[0.48]{X/{0.19}}\times5$ for $0.195\leq X\leq1.528$, where X is the OD value at 450 nm and C indicates the rhBMP2 concentration in pg/mL. The final concentrations of S1, S2, S3, S4, S5, and S6 were further calculated (refer to Figure S8).

**2.1 In *vivo* osteogenic efficacy**

This study utilized SPF-grade female SD rats aged 5-6 months, with body weights ranging from 250 to 280 grams. All animals were housed under standard conditions, with ad libitum access to autoclaved food and distilled water. The research protocol was approved by the Ethical Committee of Guangdong Second Provincial General Hospital (Project Number: 2024-DW-KZ-094-02), and all animal work guidelines were strictly followed throughout the experiment. Prior to surgery, rats were anesthetized with 5% isoflurane gas, and a 2.0 cm skin incision was made laterally along the tibia in the lower leg. Subsequently, blunt dissection with forceps was performed to expose the tibia, and a 1.5 mm Kirschner wire was drilled perpendicularly into the marrow cavity at the mid-upper segment of the tibia using an electric burr, with a depth of approximately 5 mm. After the establishment of the bone defect model, rats were randomly assigned to the control group, the ADSC-sEVs group, and the ADSC-sEVs^+^ group. Matrigel was used as a carrier for ADSC-sEVs and ADSC-sEVs^+^. In the ADSC-sEVs and ADSC-sEVs^+^ groups, 4 μL of Matrigel containing 1.5 μg/μL ADSC-sEVs and ADSC-sEVs^+^ were injected into the bone defect cavity, while the control group received an equal volume of Matrigel alone. Three weeks after surgery, rats were euthanized under deep anesthesia achieved by inhalation of carbon dioxide, and tibial samples were collected for further imaging and histological assessment.

**2.2 Microcomputed Tomography (Micro-CT) Analysis**

To quantify the ingrowth of mineralized tissue within the bone tunnel, micro-CT analysis was conducted using a high-resolution micro-CT system (NEMO, China). A total of four samples per group (n = 4) were collected for micro-CT examination three weeks following surgical intervention. The scanning parameters were optimized at an energy setting of 80 kV and an intensity of 600 μA, with a scan resolution of 35 μm. The scanning was oriented perpendicular to the longitudinal axis of the harvested bone tissue, encompassing the entire length of the bone tunnel from entry to exit points. A customized cylindrical region of interest (ROI), measuring 1.5 mm in diameter and 4 mm in height, encapsulating the bone tunnel, was defined for the quantitative assessment of newly mineralized tissue development. The ROI was reconstructed using Mimics Research software, version 21.0, to calculate and determine the bone volume (BV, in mm³), bone volume fraction (BV/TV), and tissue mineral content (TMC).

**2.3 Histological staining**

Post-micro-CT analysis, tibial samples (n = 4 per group) were fixed in 4% paraformaldehyde for 7 days and decalcified in 0.5 M EDTA solution (Biochannel, China) for 4 weeks. Dehydration was performed through a graded ethanol series (70-100% v/v) for 12 hours per step, followed by dimethylbenzene clearing and paraffin embedding. Sections were cut parallel to the bone tunnel's long axis using a saw microtome (Leica 2500E, Germany), then ground and polished to 5 μm thickness with an Exakt Micro-Grinding System (Leica, Germany). Histological evaluation was conducted on the stained sections using hematoxylin and eosin (H&E) and Goldner's trichrome staining.

**2.4 Immunofluorescent staining**

This study employed immunofluorescence staining to evaluate the impact of various interventions on the expression of osteogenic differentiation proteins in BMSCs. Following fixation, blocking, and permeabilization, the cells were incubated with specific primary antibodies (anti-rat antibodies targeting RUNX2 and OCN, obtained from Servicebio, China) overnight at 4°C. Subsequently, the cells were incubated with Cy3-conjugated goat anti-rabbit IgG secondary antibodies (Servicebio, China) for two hours at room temperature to facilitate fluorescent labeling. The cytoskeleton was stained using the phalloidin fluorescence staining method, and the nuclei were stained with DAPI. Ultimately, the immunofluorescence-stained cell sections were observed and documented using a confocal microscope (Nikon, Japan).

**2. Abbreviations**

ADSCs, adipose-derived stem cells; sEVs, small extracellular vesicles; lEVs, large extracellular vesicles; ADSC-sEVs, sEVs from adipose-derived stem cells; ADSC-sEVs^+^, sEVs from osteogenically induced adipose-derived stem cells; BMSCs, bone marrow stem cells; OPN, osteopontin; OCN, osteocalcin; RUNX2, runt-related transcription factor 2; COL1, collagen type I; ALP, alkaline phosphatase; ARS, alizarin red S; NFAPc1, nuclear factor of activated T-cells c1; GAPDH, glyceraldehyde 3-phosphate dehydrogenase; qRT-PCR, quantitative real-time polymerase chain reaction; TEM, transmission electron microscopy; NTA, nano tracking analysis; BSP, bone sialoprotein; DMEM, Dulbecco’s Modified Eagle’s Medium; PBS, phosphate buffered saline; TBST, tris-buffered saline tween-20; FBS, fetal bovine serum; BSA, bovine serum albumin; RIPA, Radioimmunoprecipitation assay buffer; PMSF, Phenylmethyl sulfonyl fluoride; DAPI, 4', 6-diamidino-2-phenylindole; GW4869, 3, 3'-(1, 4-phenylene)bis [N-[4-(4, 5-dihydro-1H-imidazol-2-yl)phenyl]-dihydrochloride-2-propenamide.

**3. Supplementary Figure Legends**

**
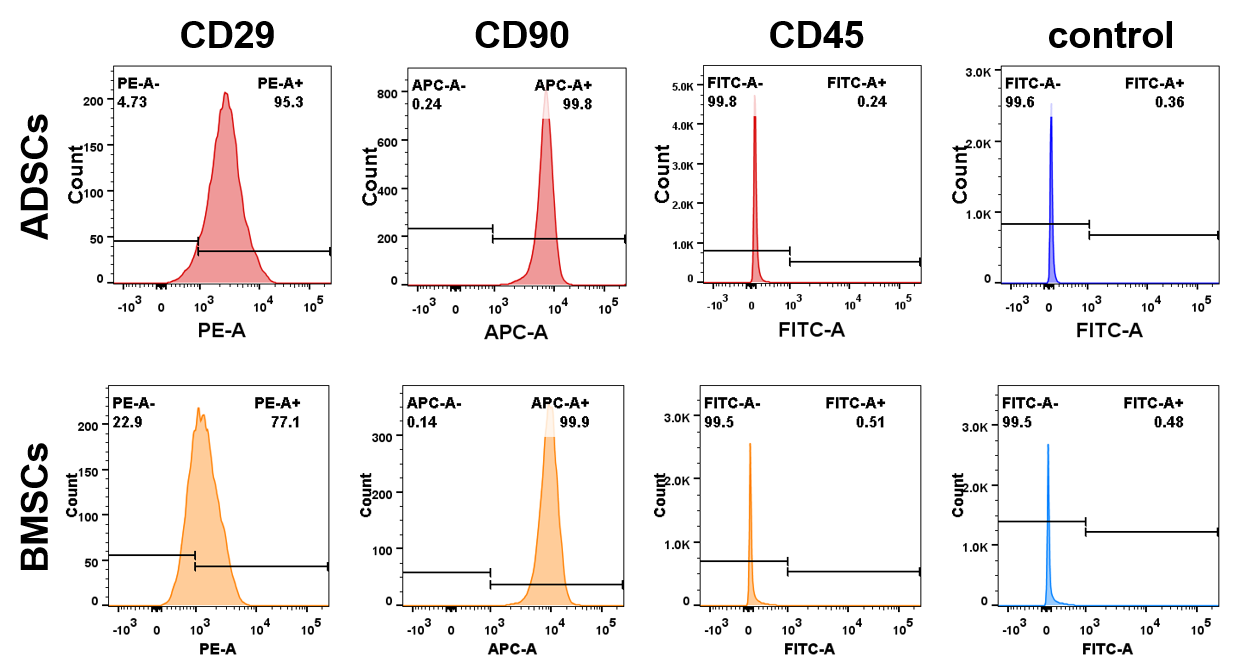
**

**Figure S1.** Flow cytometric characterization of rat ADSCs and BMSCs. The positive rates for CD29 and CD90 in ADSCs and BMSCs were as high as 95.3% and 77.1%, and 99.8% and 99.9%, respectively, whereas CD45 protein expression was negative in both cell types. BMSCs, bone marrow stem cells; ADSCs, adipose mesenchymal stem cells

**
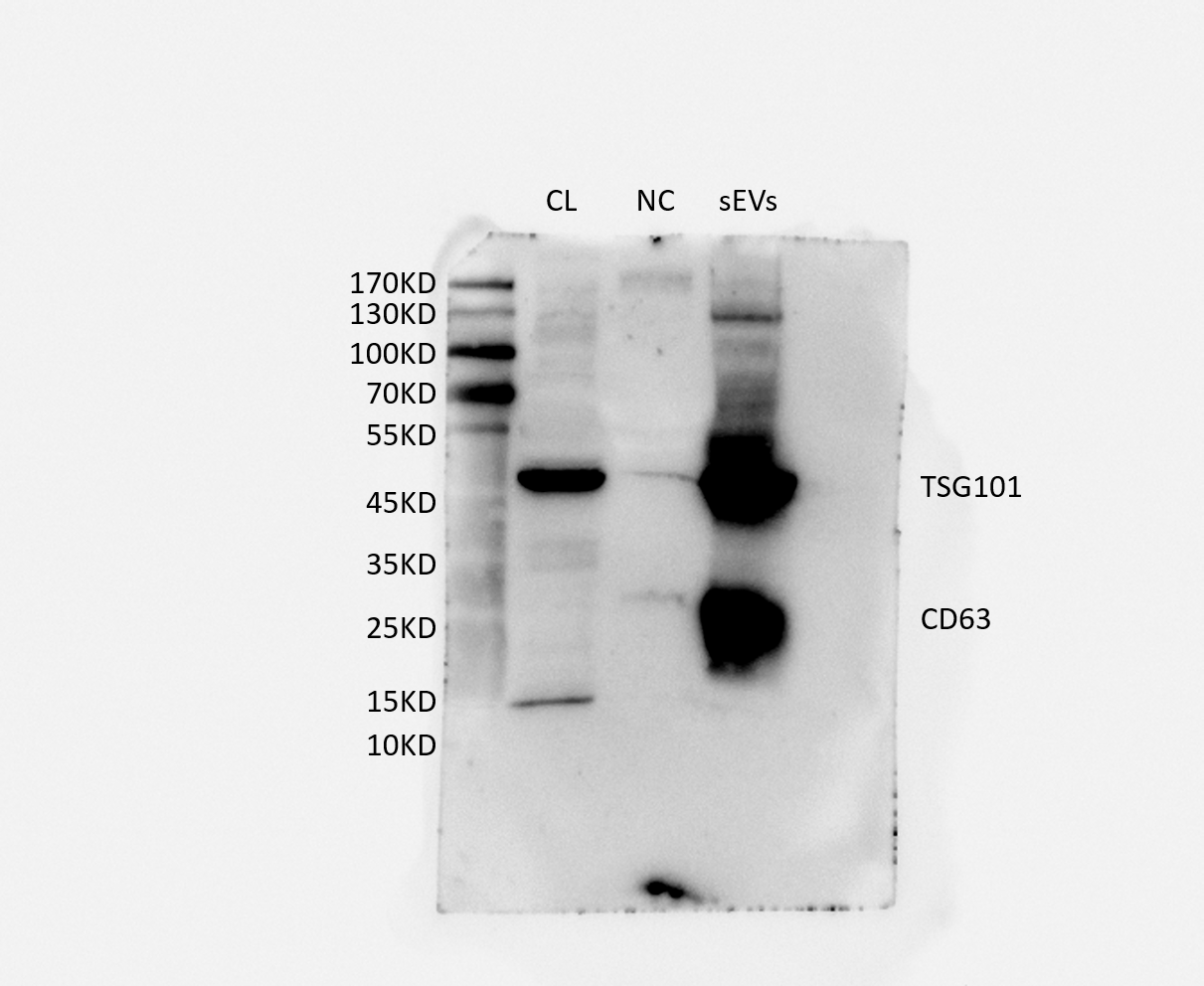
**

**Figure S2.** Western blot analysis of ADSCs lysate (CL), PBS as a negative control (NC), and sEVs for the detection of endosomal protein markers CD63 and TSG101, corresponding to the findings presented in Figure 2D. BMSCs, bone marrow stem cells; ADSCs, adipose mesenchymal stem cells; PBS, phosphate-buffered saline

**
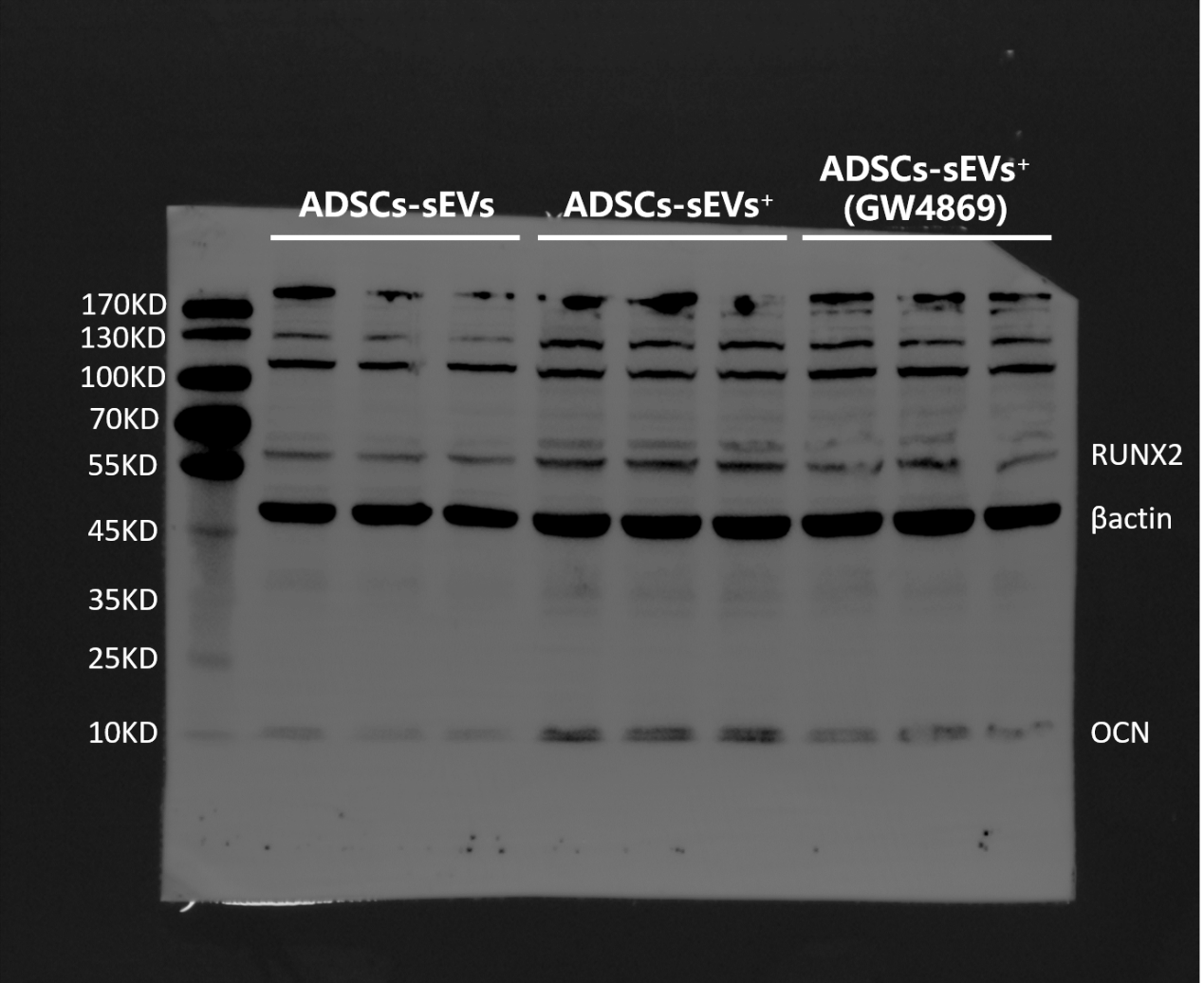
**

**Figure S3.** Western blot analysis depicting the expression of RUNX2 and OCN in BMSCs following exposure to ADSC-sEVs, ADSC-sEVs^+^, and ADSC-sEVs^+^ (GW4869), corresponding to the findings presented in Figure 4B. BMSCs, bone marrow stem cells; sEVs, small extracellular vesicles; ADSCs, adipose mesenchymal stem cells; OPN, osteopontin; OCN, osteocalcin; RUNX2, runt-related transcription factor 2

**
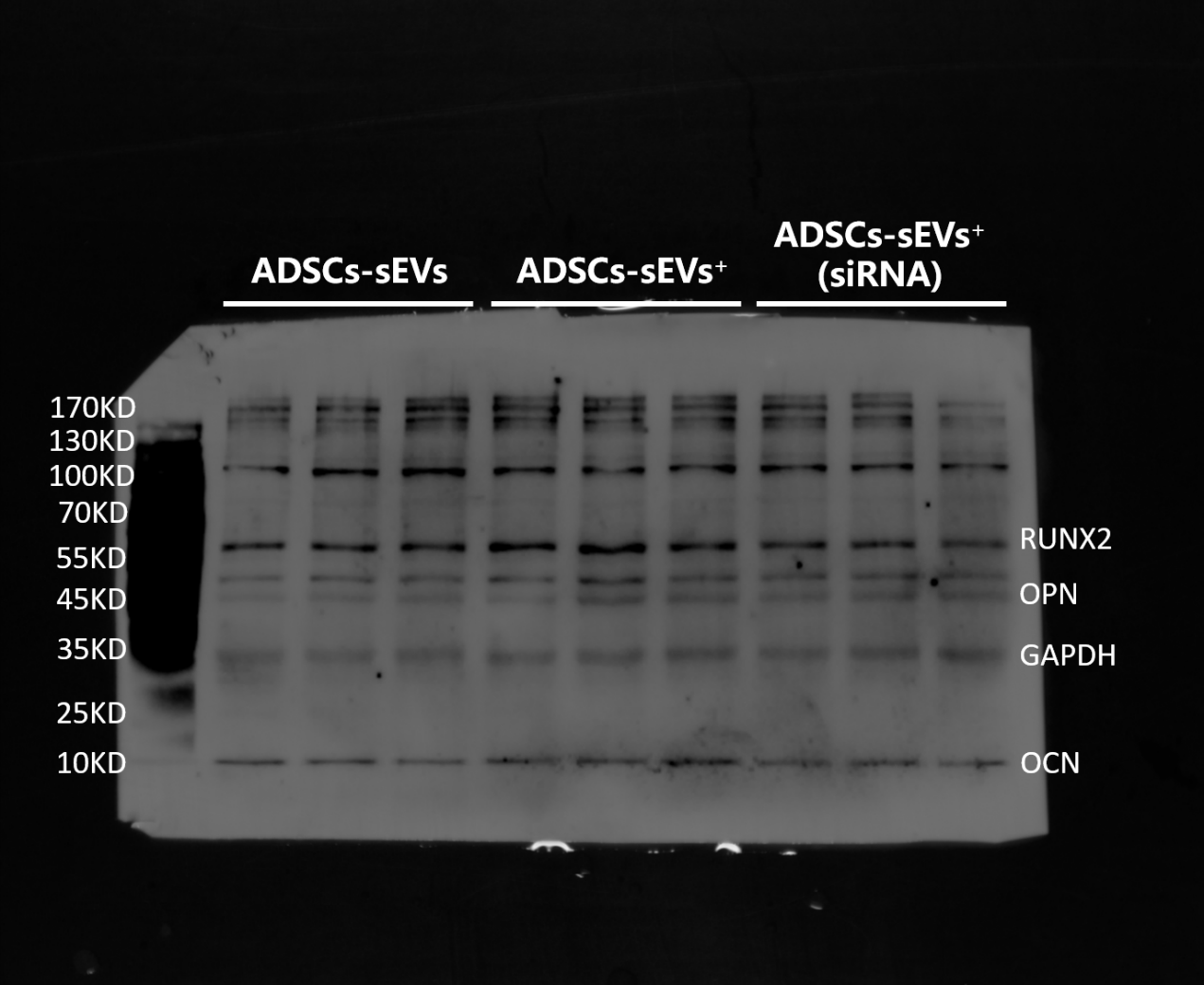
**

**Figure S4.** Western blot analysis of OPN, RUNX2, and OCN protein expression in BMSCs following exposure to ADSC-sEVs, ADSC-sEVs^+^, and ADSC-sEVs^+^ (siRNA), which is related to Figure 7D. BMSCs, bone marrow stem cells; sEVs, small extracellular vesicles; ADSCs, adipose mesenchymal stem cells; OPN, osteopontin; OCN, osteocalcin; RUNX2, runt-related transcription factor 2


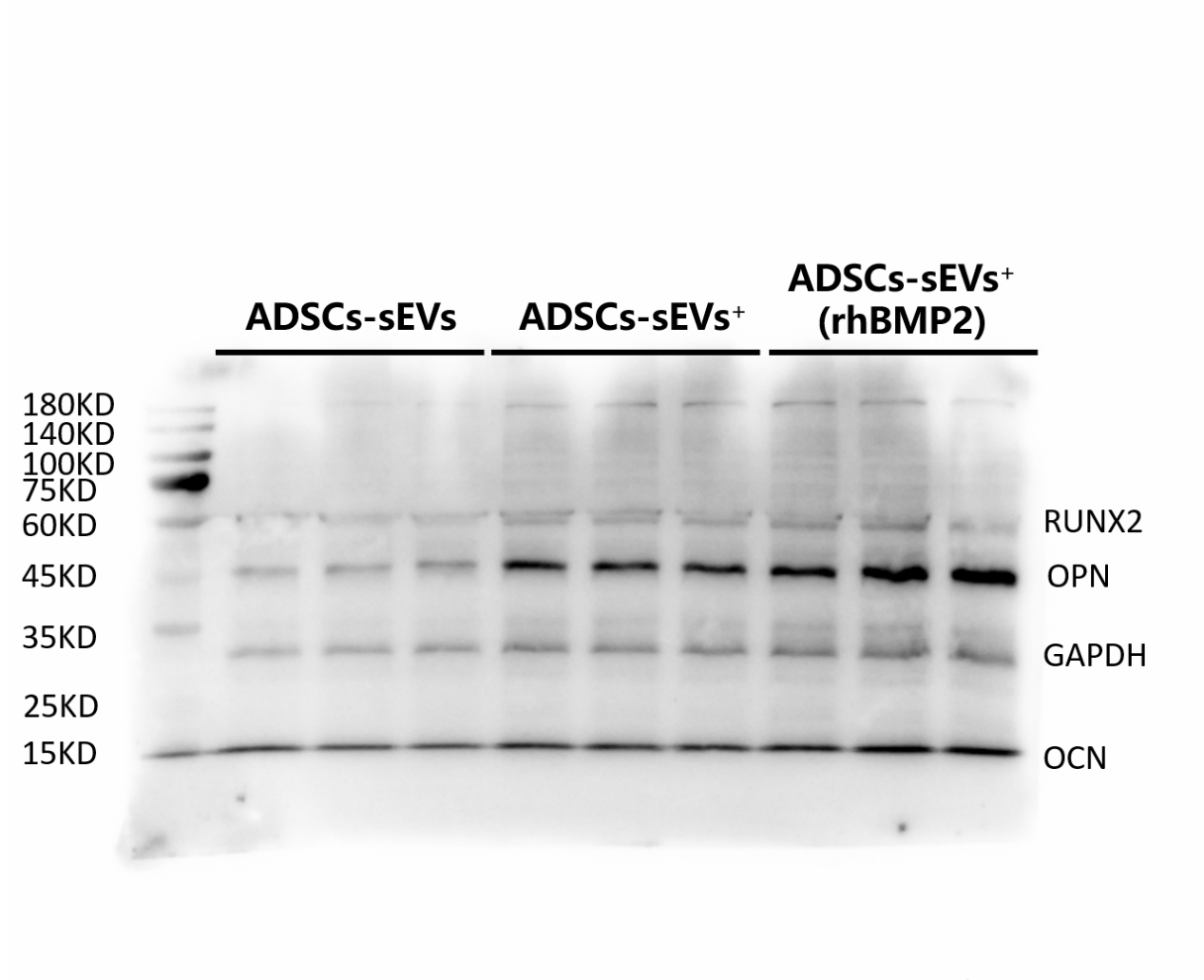


**Figure S5.** Western blot analysis of OPN, RUNX2, and OCN protein expression in BMSCs following exposure to ADSC-sEVs, ADSC-sEVs^+^, and ADSC-sEVs^+^ (rhBMP2), which is related to Figure 8F. BMSCs, bone marrow stem cells; sEVs, small extracellular vesicles; ADSCs, adipose mesenchymal stem cells; OPN, osteopontin; OCN, osteocalcin; RUNX2, runt-related transcription factor 2

**
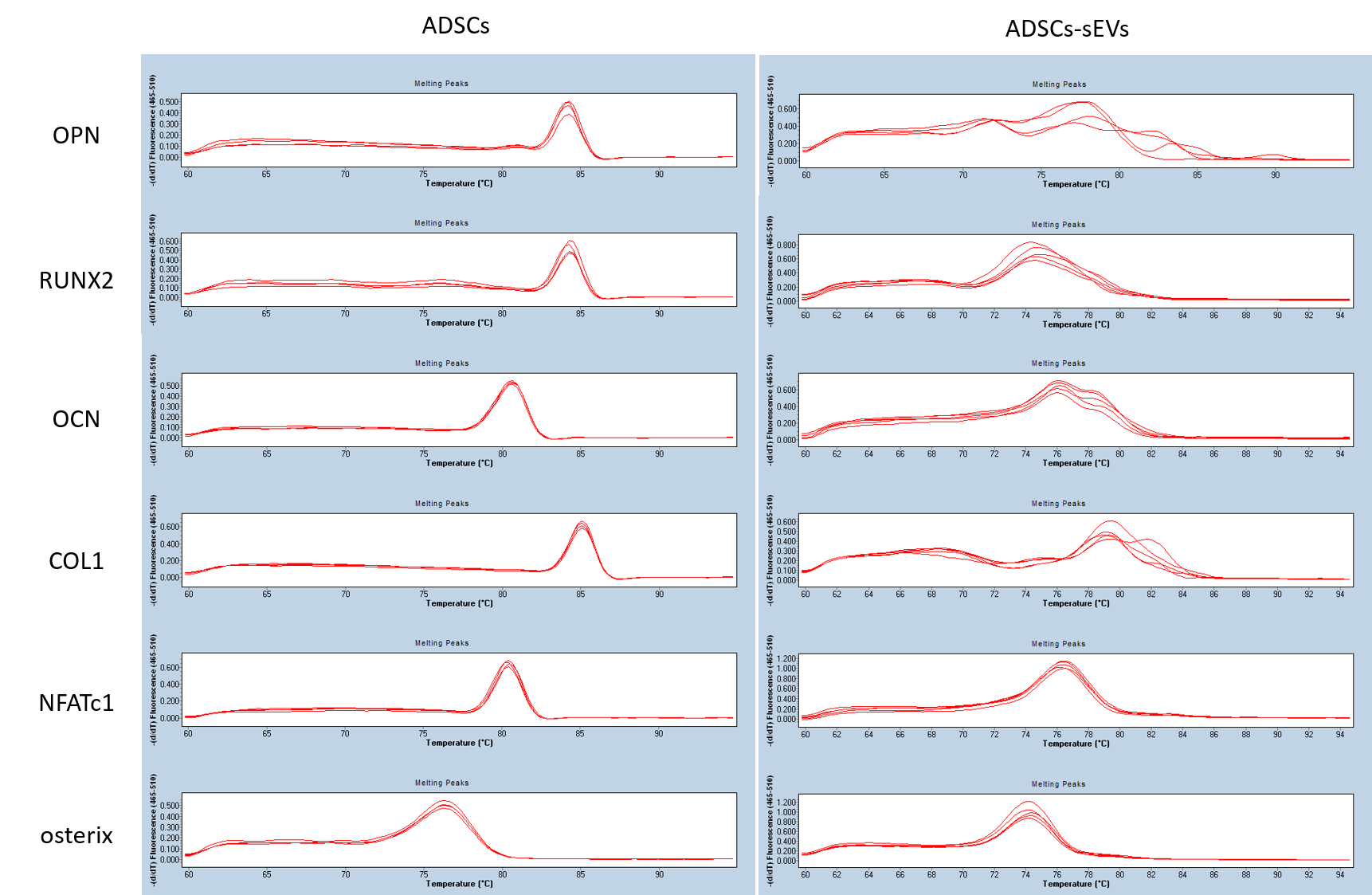
**

**Figure S6.** Melting curves of osteogenically related mRNAs during the PCR process. All mRNAs in ADSCs exhibited distinct and singular peak melting curves, whereas osterix and NFATc1 displayed single peak melting curves in ADSC-sEVs, whereas other mRNAs demonstrated complex multi-peak melting curves. (n = 5). sEVs, small extracellular vesicles; ADSCs, adipose mesenchymal stem cells; NFATc1, and nuclear factor of activated T-cells c1


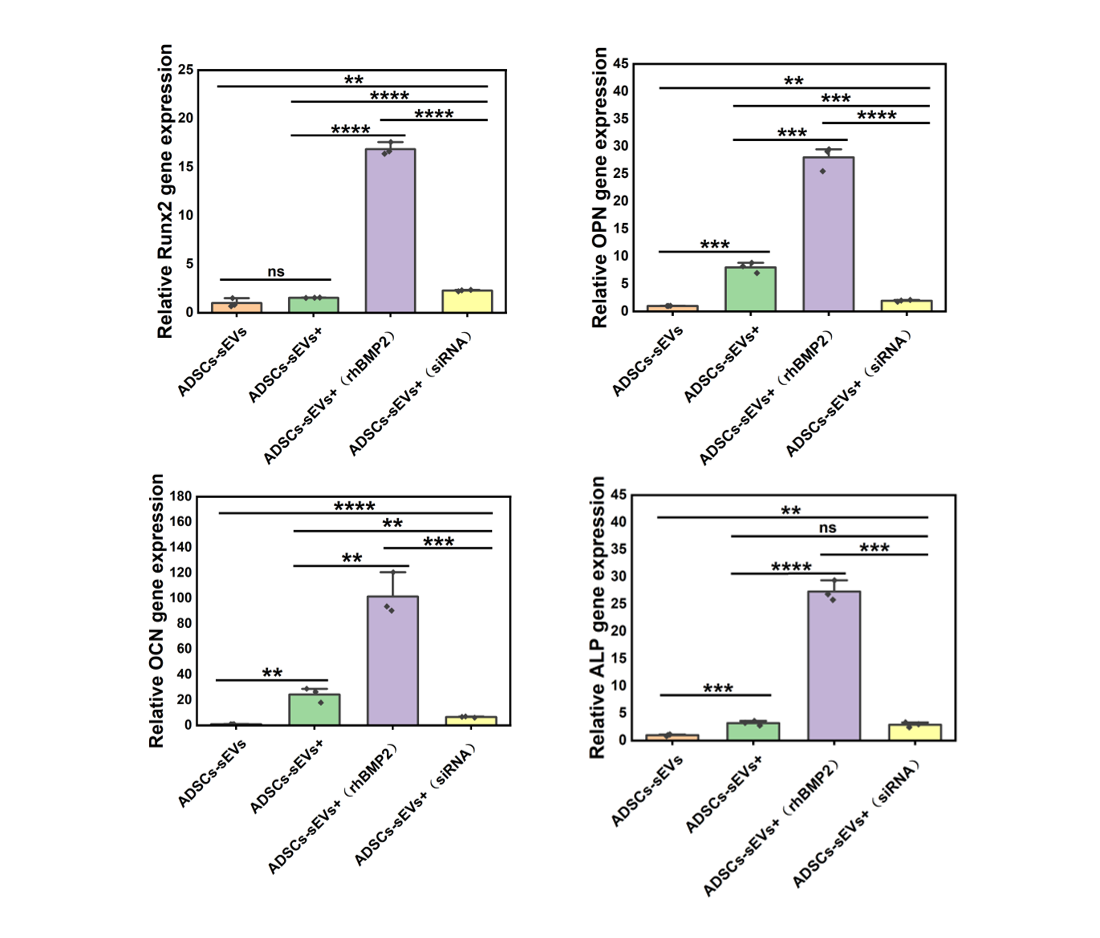


**Figure S7.** The expression of the osteogenosis-related gene (RUNX2, OPN, OCN and ALP) in rabbit BMSCs cocultured with different samples for 7 days. (n = 3, ***P* < 0.01, ****P* < 0.001, *****P* < 0.0001, ns = not significant). BMSCs, bone marrow stem cells; OPN, osteopontin; OCN, osteocalcin; RUNX2, runt-related transcription factor 2; ALP, alkaline phosphatase


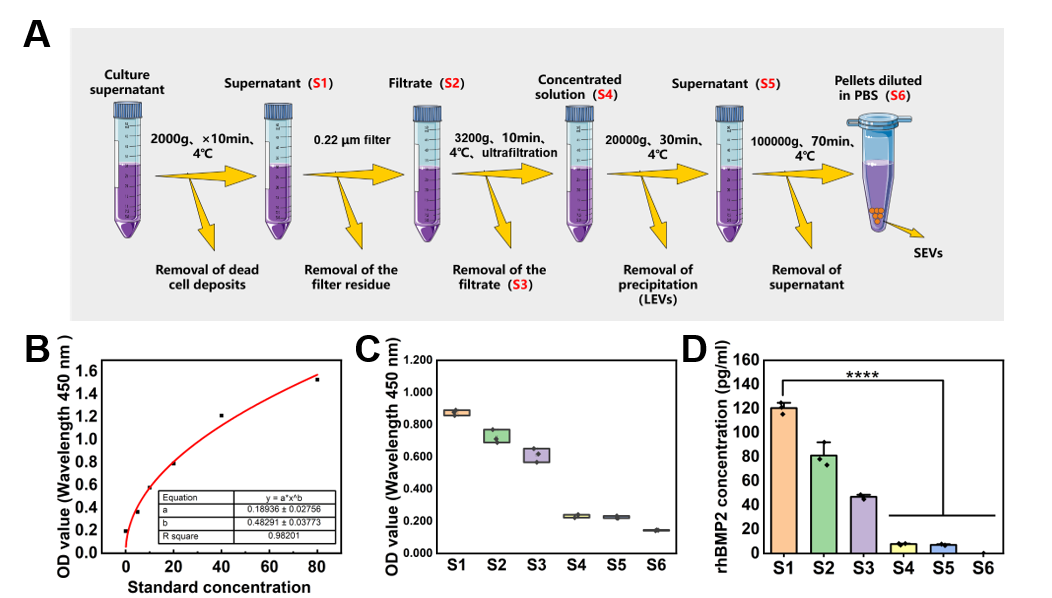


**Figure S8.** Purification process, and rhBMP2 contamination assessment of ADSC-sEVs^+^ (rhBMP2). **A:** Schematic overview of the ADSC-sEVs purification protocol. **B:** ELISA standard curve for rhBMP2, indicating the calibration range and sensitivity of the assay. **C and D:** Concentration measurements of residual rhBMP2 in ADSC-sEVs^+^ (rhBMP2) samples across different preparation stages, as determined by ELISA. (n = 3, *****P* < 0.0001). sEVs, small extracellular vesicles; ADSCs, adipose mesenchymal stem cells; ELISA, enzyme-linked immunosorbent assay

**4. Supplementary Tables**

**Table S1. Antibodies for Flow Cytometry, Western Bolt and Immunofluoresence Application**

| **Antigen** | **Host** | **Application** | **Manufacture** | **Product code** |
| --- | --- | --- | --- | --- |
| CD29 PE | Mouse | Flow Cytometry | RD | MAB17782-SP |
| CD29 PE | Hamster | Flow Cytometry | Elabscience | E-AB-F1309D |
| CD90 FITC | Mouse | Flow Cytometry | ThermoFisher | MA1-81491 |
| CD90 APC | Mouse | Flow Cytometry | Elabscience | E-AB-F1226E |
| CD105 PerCP | Mouse | Flow Cytometry | BD Biosciences | 560819 |
| CD44 FITC | Rat | Flow Cytometry | Genetex | GTX15883 |
| CD34 FITC | Rat | Flow Cytometry | Genetex | GTX28158 |
| CD45 FITC | Mouse | Flow Cytometry | Genetex | GTX628507 |
| CD63 | Rabbit | Western Bolt | Abcam | ab134045 |
| TSG101 | Mouse | Western Bolt | Abcam | AB133586 |
| OCN | Mouse | Western Bolt | ThermoFisher | 33-5400 |
| OPN | Mouse | Western Bolt | RD | NB110-89062 |
| RUNX2 | Rabbit | Western Bolt | Bioss | bs-1134R |
| βactin | Mouse | Western Bolt | Santa Cruz | sc-47778 |
| GAPDH | Rabbit | Western Bolt | ThermoFisher | PA5-85074 |
| RUNX2 | Rabbit | Immunofluoresence | Servicebio | GB115631 |
| OCN | Rabbit | Immunofluoresence | Servicebio | GB115684 |
| Cy3 | Goat | Immunofluoresence | Servicebio | GB21303 |

**Table S2. Sequences of siRNA**

| **siRNA template** | **Target sequences** |
| --- | --- |
| **si-ocu-SP7_001** | CTTACGGCTCCTGGTACAA |
| **si-ocu-SP7_002** | CTGCTCAACCTCCACTGAA |
| **si-ocu-SP7_003** | GAGTGACCACCTGAGCAAA |

**Table S3. Primer sequence**

| **RNA template** | **Gene ID** | **Primer sequences** |
| --- | --- | --- |
| **OPN** | 100008982 | AACAAGAGACCCTCCCGAGT |
|  |  | TCGGCATCGTCGGATTCATT |
| **OCN** | 100009355 | TGGATGGTTTGTTGTTCTGC |
|  |  | AGGTGGTGATGGGGTTACAA |
| **RUNX2** | 100008943 | TCCGAAATGCCTCTGCTGTT |
|  |  | CGGGGTCCATCCACTGTAAC |
| **COL1** | 100340475 | GCCTCTCTCTGGCAGGAAAC |
|  |  | ATGATCTCCGTCTGTGCGTG |
| **NFATc1** | 100352378 | CGTTCTCTCCAACACCAAGG |
|  |  | CTTCTCCACAAGGGGCAGTT |
| **Osterix** | 100339510 | TCAACCTCCACTGAACCCC |
|  |  | CCTGGTTGTAGGAGGTGGGG |
| **GAPDH** | 100009074 | GTGAAGGTCGGAGTGAAC |
|  |  | GGTGGAATCATACTGGAACA |
| **U6** | 100356871 | TGCTCTCAGTCTCCGAGTGTCAG |
|  |  | TGTGTGGCTCCTCCTTTCCTCTC |
| **RUNX2** | [367218](https://www.ncbi.nlm.nih.gov/gene/367218) | ACTTCCTGTGCTCGGTGCT |
|  |  | GACGGTTATGGTCAAGGTGA |
| **OPN** | [25353](https://www.ncbi.nlm.nih.gov/gene/25353) | AGCAAGAAACTCTTCCAAGCAA |
|  |  | GTGAGATTCGTCAGATTCATCCG |
| **OCN** | [690625](https://www.ncbi.nlm.nih.gov/gene/690625) | GCTCAGTCTGGCATCAACTCC |
|  |  | CTGGTATCTGTGGGCTTTTCAG |
| **ALP** | [25586](https://www.ncbi.nlm.nih.gov/gene/25586) | AACGTGGCCAAGAACATCATCA |
|  |  | TGTCCATCTCCAGCCGTGTC |

**Table S4. Information of the EVs used for subsequent experiment**

| **EVs types** | **Protein concentration**  **(μg/μL)** | | **Total RNA (ng/μL)** | **A260/A280** | | **Survival rate of source cells** |
| --- | --- | --- | --- | --- | --- | --- |
| **Rabbit-ADSC-sEVs** | 1.446 | 31.6 | | | 1.77 | 99.98% |
| **Rabbit-ADSCs-lEVs** | 1.544 | 29.6 | | | 1.79 | 99.98% |
| **Rat-ADSC-sEVs** | 1.573 | 24.8 | | | 1.21 | 99.92% |
| **Rabbit-ADSC-sEVs(GW4869)** | 0.467 | 14.7 | | | 1.14 | 95.42% |
| **Rabbit-ADSC-sEVs(siRNA)** | 1.868 | 36.3 | | | 1.24 | 70.75% |
| **Rabbit-ADSC-sEVs(rhBMP2)** | 3.392 | 56.9 | | | 1.49 | 99.92% |

**Table S5. Quantitative micro-CT analysis of bone defect in NC, ADSC-sEVs and ADSC-sEVs+ groups**

| **group** | **BV(mm^3)** | **TV(mm^3)** | **BV/TV** | | **BS/TV (mm^-1)** | **Connectivity** | **TMC(mg)** |
| --- | --- | --- | --- | --- | --- | --- | --- |
| **NC** | 1.516 ± 0.58 | 8.836 ± 0.02 | 0.172 ± 0.06 | 2.194 ± 0.60 | | 35.750 ± 29.62 | 2.098 ± 0.81 |
| **ADSC-sEVs** | 2.295 ± 0.06 | 8.831 ± 0.02 | 0.260 ± 0.01 | 3.277 ± 0.30 | | 85.250 ± 20.43 | 3.166 ± 0.08 |
| **ADSC-sEVs^+^** | 3.097 ± 0.25 | 8.848 ± 0.01 | 0.350 ± 0.03 | 4.342 ± 1.09 | | 115.00 ± 68.68 | 4.448 ± 0.16 |
| **Data were expressed as mean ± S.D. (*n* = 4)** | | | | | | | |
